# Methyl pyruvate protects a normal lung fibroblast cell line from irinotecan-induced cell death: Potential use as adjunctive to chemotherapy

**DOI:** 10.1371/journal.pone.0182789

**Published:** 2017-08-10

**Authors:** Bernice Monchusi, Monde Ntwasa

**Affiliations:** 1 School of Molecular & Cell Biology, Gatehouse 514, University of the Witwatersrand, Wits, Johannesburg, Republic of South Africa; 2 Department of Life & Consumer Sciences, 211 Calabash Building, University of South Africa, Florida, Johannesburg, Republic of South Africa; University of South Alabama Mitchell Cancer Institute, UNITED STATES

## Abstract

The Warburg Effect, characterized by increased rate of glycolysis even under normoxic conditions, is one of the hallmarks of cancer. Relatively lower oxidative phosphorylation (OXPHOS) is also a characteristic feature in cancer cells. We hypothesized that interference with this phenomenon, by introducing exogenous pyruvate, would upset this cancer phenotype and boost the energy requirements of normal cells. We find that methyl pyruvate protects irinotecan-treated normal lung fibroblast cell line (MRC-5) probably by turning off the p53/p21 axis of the apoptotic pathways. When the MRC-5 fibroblasts recover in drug-free medium, the intrinsic apoptotic pathway is also turned off and the cells survive with no discernible exponential growth during the observation period. In contrast, the mere introduction of exogenous pyruvate kills the lung cancer cell line (A549). Although, functional p53 is important in the drug-induced cancer cell death, it is probably not essential because cancer cell lines with mutated p53 also die albeit less efficiently. We conclude that methyl pyruvate may preferentially kill cancer cells and protect normal cells during chemotherapy.

## Introduction

Metabolic reprogramming, also known as the Warburg Effect, is one of the hallmarks of cancer [1]. Otto Warburg discovered that cancer cells undergo metabolic reprogramming with concomitant increase in glucose uptake. Primarily, they utilize the glycolytic pathway to metabolize glucose even in normoxic conditions [2]. This phenomenon led to opportunities for drug discovery and development.

Otto Warburg noticed that while normal and tumour cells could undergo glycolysis in the presence of oxygen, normal cells died and cancer cells continued to thrive. It is now recognized that reduced oxidative phosphorylation (OXPHOS) is a characteristic feature in cancer cells [3–5]. It is likely that by enhanced glycolysis cancer cells do not invest primarily in ATP production but to anabolic processes such as lipid synthesis and *de novo* nucleic acid synthesis [6,7]. Firstly, cells stimulated to use aerobic glycolysis always maintain high ATP:ADP and NADH:NAD^+^ ratios; with high levels of glycolysis- derived NADH [8]. These ratios are important to prevent cells undergoing apoptosis due to ATP depletion. Secondly, certain growth factors cause cells to release fructose-1,6-bisphosphate, the allosteric activator of pyruvate kinase, leading to diversion of glucose metabolites from energy production to anabolic processes [9].Cancer cells have an obvious priority to increase biomass as they have to double their cell mass. This can be done by promoting anabolic activities via the pentose phosphate pathway (PPP). The main functions of the PPP are to produce NADH which is required as a reducing agent in biosynthetic reactions and the synthesis of ribulose-5-phosphate which is required for nucleotide and nucleic acid synthesis [10]. Furthermore, the glycolytic intermediates 3- phosphoglycerate and pyruvate are precursors for the biosynthesis of some non-essential amino acids and pyruvate contributes to production of the other non-essential amino acids via precursors in the tricarboxylic acid cycle (TCA) [6].

The tumor suppressor protein, p53 is often the target of chemotherapeutic drugs due to its central role in the maintenance of cellular integrity and protection against DNA damage [11]. In recent years, it emerged that p53 also controls glycolysis and oxidative respiration thus underlining its crucial influence on glucose metabolism [12,13]. Overall, p53 inhibits glycolysis at key points and promotes respiration. In the first instance, p53 regulates glucose and glutamine uptake by modulating the functions of glucose transporters such as GLUT1-4. Once glucose is inside the cell, p53 activates TIGAR (TP53-induced glycolysis and apoptosis regulator) which inhibits the conversion of fructose-6-phosphate to fructose 2,6-bisphosphate thereby diverting glycolysis to the pentose phosphate pathway (PPP). p53 can also boost the PPP by promoting the conversion of glucose–6-phosphate into ribose-5-phosphate advancing nucleotide synthesis. Further downstream the glycolytic pathway, p53 directly inhibits transcription of the phosphoglycerate mutase (PGM) gene [14,15]. Normal cells sense glucose availability using a p53 dependent pathway whereby the AMP-activated protein kinase phosphorylates p53 on serine 15 resulting in arrest of the cell cycle at the G1/S boundary [16]. These findings imply that p53 regulators such as Mouse Double Minute (Mdm2) and the retinoblastoma binding protein 6 (RBBP6) also influence glucose metabolism.

The discovery that p53 regulates the cytochrome c oxidase complex, a major element of complex IV of the mitochondrial electron transport chain (ETC), underscores the importance of p53 in modulating OXPHOS. This is achieved by transcriptional control of the Synthesis of Cytochrome c Oxidase 2 (SCO2) gene. In the absence of p53, cells shift glucose metabolism to the glycolytic pathway and reduce respiration. A similar outcome is obtained when the *SCO2* gene is disrupted, a phenotype that is rescued when SCO2 function is restored. This phenotype rescue occurs even in a p53-deficient background [17]. Supporting evidence for the Warburg Effect directing glucose metabolism mainly towards anabolic processes rather than of ATP production is that overall ATP production is the same in all wild-type, heterozygous and null p53-backgrounds as the shift to glycolysis is balanced by proportional oscillations in OXPHOS-generated ATP [13,17]. These experiments also reaffirm the crucial role of p53 in modulating the rates of glycolysis and oxidative phosphorylation.

It is expected that the differential metabolic programmes of normal and cancer cells provide the basis for development of cancer-specific therapeutic strategies. Due to the harshness of chemotherapy, it is crucial to find strategies that selectively protect normal cells. In the current work we show that introduction of exogenous pyruvate preserves normal cells and induces apoptosis in cancer cell lines. We propose that this approach could be exploited for reducing the toxic effects of chemotherapy.

## Materials and methods

### Cell culture and conditions

American Type Culture Collection (ATCC) cell lines Human lung adenocarcinoma (A549; ATCC^®^ CCL-185^TM^), breast adenocarcinoma (MDA-MB 231; ATCC^®^ CRM-HTB-26^TM^) and lung embryonic fibroblast (MRC-5; ATCC^®^ CCL-171^TM^) cell lines were obtained from ATCC. Human ovarian clear cell adenocarcinoma (RMG-1; CVCL-1662) cell line was obtained from ATCC and Japanese collection of research and bioresources (JCRB). All of the above mentioned cell lines were cultured in DMEM (Dulbecco's modified essential medium) growth media (Sigma, D6429) supplemented with 10% Fetal Bovine Serum (Sigma, F0804) and 1% penicillin/streptomycin (Sigma, P4333). MDA-MB 231 expresses mutant p53 while the others express wild type p53. All cultures were maintained at 37°C with 5% CO_2_.

### Drugs

Irinotecan (Sigma, 1347609) and methyl pyruvate (both Sigma-Aldrich) were dissolved in water.

### Dynamic monitoring of cell proliferation and cytotoxicity profiles

Cell proliferation was monitored using the *xCELLigence* Technology (ACEA Biosciences, USA) which measures electrical impedance generated by adherent cells. Cells were seeded at cell densities of 5 *x* 10^3^ for RMG-1 cells and 1 *x* 10^4^ for A549, MDA-MB 231 and MRC-5 cells into 16 well gold coated microelectrode E-plates until cell numbers reached a logarithmic phase. At pre-determined time points various combinations of 0.5 μM irinotecan, 1 mM and 2 mM methyl pyruvate were applied to the cells to determine cytotoxicity profiles. In two independent experiments, proliferation profiles of untreated and treated cells were recorded at 4, 24 and 48 42 hours. The normal fibroblast cell line (MRC-5) was further characterized for recovery profiles, also in two independent experiments. Towards this end, the medium containing the drugs was replaced with fresh DMEM supplemented with foetal bovine serum and further incubated for another 24 hours. Profiles were recorded as time versus cell index plots.

### Fluorescence-activated cell sorting (FACS) analysis

Approximately 3 x 10^6^ cells/ml of RMG-1, A549, MDA-MB 231 and MRC-5 cell lines were treated with 2 mM methyl pyruvate, 0.5 μM irinotecan or their combination for 4, 24 and 48 hours. At these time points, they were harvested using trypsin and centrifugation at 5000 x g for 15 minutes. The pellet was then washed with pre-chilled 1X PBS (phosphate-buffered saline) and re-suspended in 300 μl 1X PBS (Sigma P5493-1lL) followed by fixing with 70% ethanol. The fixative was removed by centrifugation at 5000 x g for 10 minutes. The pellets were then stained with FxCycle PI/RNase staining solution which contains propidium iodide to facilitate quantification of DNA. The samples were then incubated at room temperature in the dark for 30 minutes. FACS analysis was conducted using the BD Accuri C6 Flow Cytometer (BD Biosciences, USA).

### Annexin V FITC apoptosis assay

Cells were treated and harvested as described above. After rinsing the pellets with 1 X PBS, cells were resuspended in 1 X 10^6^ aliquots in 1 x binding buffer (Sigma B9796-1VL) and incubated for 10 minutes in the dark at room temperature with 5 μl of Annexin V-FITC (Sigma A9210) and 10 μl propidium iodide (Sigma P2667). Samples were immediately analyzed using FACS to determine the mode of cell death induced by the drugs.

### Reverse transcription polymerase chain reaction (RT-PCR)

RNA extraction was performed using the trizol RNA isolation method[18] and total RNA was quantified using the Nanodrop spectrophotometer (Thermo Fischer Scientific, California, USA). Messenger RNA was converted to cDNA using the First Strand cDNA Synthesis Kit (New England Biolabs) at 42°C for 60 minutes. The cDNA was amplified in a 50 μl reaction containing 25 μl 2 X PCR master mix, 0.2 μM forward and reverse primers and nuclease free water to make the volume. After initial denaturation for 5 minutes, the various targets namely; *BID*, *Bax*, *p21*, *caspase 3*, *MDM2* and *GAPDH* were amplified in 35 cycles as follows: 94°C for 30 seconds, 30 seconds at the appropriate annealing temperature for each primer set S1 Table and at 72°C for 1 minute. This was followed by final extension at 72°C for 5 minutes. The PCR products were separated in an agarose gel with ethidium bromide to visualize the bands using the ChemiDoc™ MP Imaging system.

### Western blot analysis

Proteins were extracted from the cell lines in RIPA buffer (50 mM Tris-HCl, pH 8.0, 150 mM sodium chloride, 1.0% NP-40 (Non-ident P 40), 0.5% sodium deoxycholate, and 0.1% sodium dodecyl sulfate) after incubation for 4, 24 and 48 hours. Subsequently, they were separated by the Towbin method [19] using 12% separating gel and 4% stacking gel with the exception of the RBBP6 protein which was separated on 8% separating and 4% stacking gels to overcome difficulties caused by their high overall positive charge [20]. The proteins were then transferred onto a PVDF membrane (Amersham Hybond-P) soaked in Towbin buffer using the Hoefer VE mini system at 70 V for 90 minutes except for RBBP6, which was transferred overnight. All membranes were blocked using SuperBlock^®^ (PBS) Blocking solution (Thermo Scientific) for 30 minutes and then washed with 1X PBS- Tween 5 times for 5 minutes per wash. The membranes were then probed with the following primary antibodies overnight: anti-p53 monoclonal ab28 (1:500) (PAb 1801, Abcam), anti-RBBP6 rabbit polyclonal (1:500) (NBPI-49535, Novus Biologicals), anti-cytochrome c goat polyclonal (1:1000) (Sc-7159, Santa cruz) and anti-actin monoclonal (1:500) (Sc-8432, Santa cruz) all diluted in 1X PBS Tween. This was followed by washing with 1X PBS Tween 5 times for 5 minutes per wash. The secondary antibodies; anti-mouse IgG (1:5000) (Sc-2005, Santa-Cruz), anti-rabbit l IgG (1:5000) (A8919, Sigma), anti-goat IgG (1:5000) (A-0545, Sigma) and anti-mouse IgG (1:1000) (Sc-2005, Santa cruz) were applied for 1 hour at 37°C. The membranes were then washed five times with 1X PBS Tween for 5 minutes per wash. 2 ml SuperSignal® West Pico Chemiluminescent Substrate (Thermo Scientific) was then added to each blot in a 1:1 ratio (Lumino/Enhancer and Stable peroxide buffer) followed by a 5 minute incubation for in the dark. The signals were recorded using the Gel Doc XR^+^ imaging system (170-S170, Bio Rad).

### RT^2^ Profiler PCR array analysis

DNAse-free total RNA was extracted from A549 and MRC-5 cells using the RNeasy Kit (Qiagen). RNA was quantified using the Nanodrop and converted to cDNA as described by the manufacturer (QIAGEN-330401).

The Human Cancer PathwayFinder^TM^ RT^2^ Profiler^TM^ PCR array (QIAGEN- PAHS-033ZF-12) microarray profiles the expression of 84 genes involved in 9 biological pathways that mediate carcinogenesis. Four microarrays were set up to determine differential gene expression in the lung cancer cell line (A549) and in the normal lung fibroblast cell line (MRC-5). Expression was measured in untreated cells and in cells treated for 48 hours with both irinotecan and methyl pyruvate for each cell line. A 27000 μl reservoir of the cDNA amplification reaction containing 1350 μl of the 2 x RT^2^ SYBR Green Mastermix (QIAGEN- 330500), 102 μl cDNA synthesis reaction mix, and 1248 μl RNase-free water (QIAGEN-129117) was prepared. Using the INTERGA VIAFLO ASSIST liquid handler (Interga Biosciences—CH-7205 Zizers) 25 μl aliquots were distributed into the 86 well microplate, each well containing pre-dispensed gene-specific primer sets. This was followed by centrifugation at 1000 x g for 1 minute at room temperature using the low speed benchtop centrifuge (Grant instruments—LCM-3000). After initial heating step to activate the HotStart DNA Taq polymerase for 10 minutes at 95° C were amplified in 45 cycles as follows: 95° C for 15 seconds and 60° C for 1 minute respectively, using the real-time light cycler (Roche LC-480).

### Differential gene expression in response to treatment by a combination of methyl pyruvate and irinotecan

Following each run, the threshold cycle (C_T_) for each well was calculated using the Roche LightCycler 480 software. Differentially expressed genes were identified using the QIAGEN data analysis web portal. C_T_ values were normalized based on selection of reference genes that showed small changes in their expression across different sample groups (differences in C_T_ < 1). The C_T_ values for these genes were geometrically averaged and used for the fold change ΔΔ C_T_ calculations. Genes with greater than twofold change in gene expression were selected.

### Pathway analysis of differentially expressed genes using the PANTHER classification system

To classify the differentially expressed genes in both A549 and MRC-5 cells and to cluster molecular interaction helping in predicting perturbed biological processes, the PANTHER classification system (http://pantherdb.org/) was used. Differentially expressed genes that are unique to A549 or MRC-5 were uploaded into PANTHER and submitted to explore biological pathways that may be potentially perturbed by the drugs.

### Network-based gene expression analysis

GeneMANIA through the Cytoscape plugin was used to determine whether changes in gene expression due to combined drug treatment, described previously in A549 and MRC-5 cells reflects possible changes in the function of certain biochemical pathways. GeneMANIA was installed through the Cytoscape plugin manager tab. All data for *Homo sapiens* (version 2014-08-12-core) was downloaded into GeneMANIA consisting of: 269 networks, 20531 genes and 14373062 interactions. The query lists containing differentially expressed genes in A549 and in MRC-5 cells were uploaded using gene name symbols. GeneMANIA displayed an interactive functional association network that extended the query gene list by including genes that were functionally related producing a relationship network from the input data set (Warde-Farly et al., 2010).

### REACTOME pathway analysis

To further delineate that perturbed biological processes the REACTOME pathway analysis tool (http://www.reactome.org) was used. Differentially expressed genes in A549 or MRC-5 cells with their associated fold change values were uploaded into REACTOME. The data were used to generate a pathway view diagram. Upregulated genes/pathways/processes are represented by bright yellow and bright blue downregulated genes/processes. Pathways were considered statistically enriched when the p < 0.05. The REACTOME database includes annotations for 7088 of the 20 774 protein-coding genes in the current Ensemble human genome assembly (34% coverage), 1421 small molecules organized into 6744 reactions collected in 1481 pathways, and 15 107 literature references [21].

### Statistical analysis

Cell cycle and cell death analyses are presented as the mean value ± SEM of at least three independent experiments. Statistical analysis was performed with the paired student *t*-test comparing two groups at a time. P-value < 0.05 was set and were considered significant.

## Results

### Methyl pyruvate protects MRC-5 fibroblasts from irinotecan induced cell death

**Fig 1:**
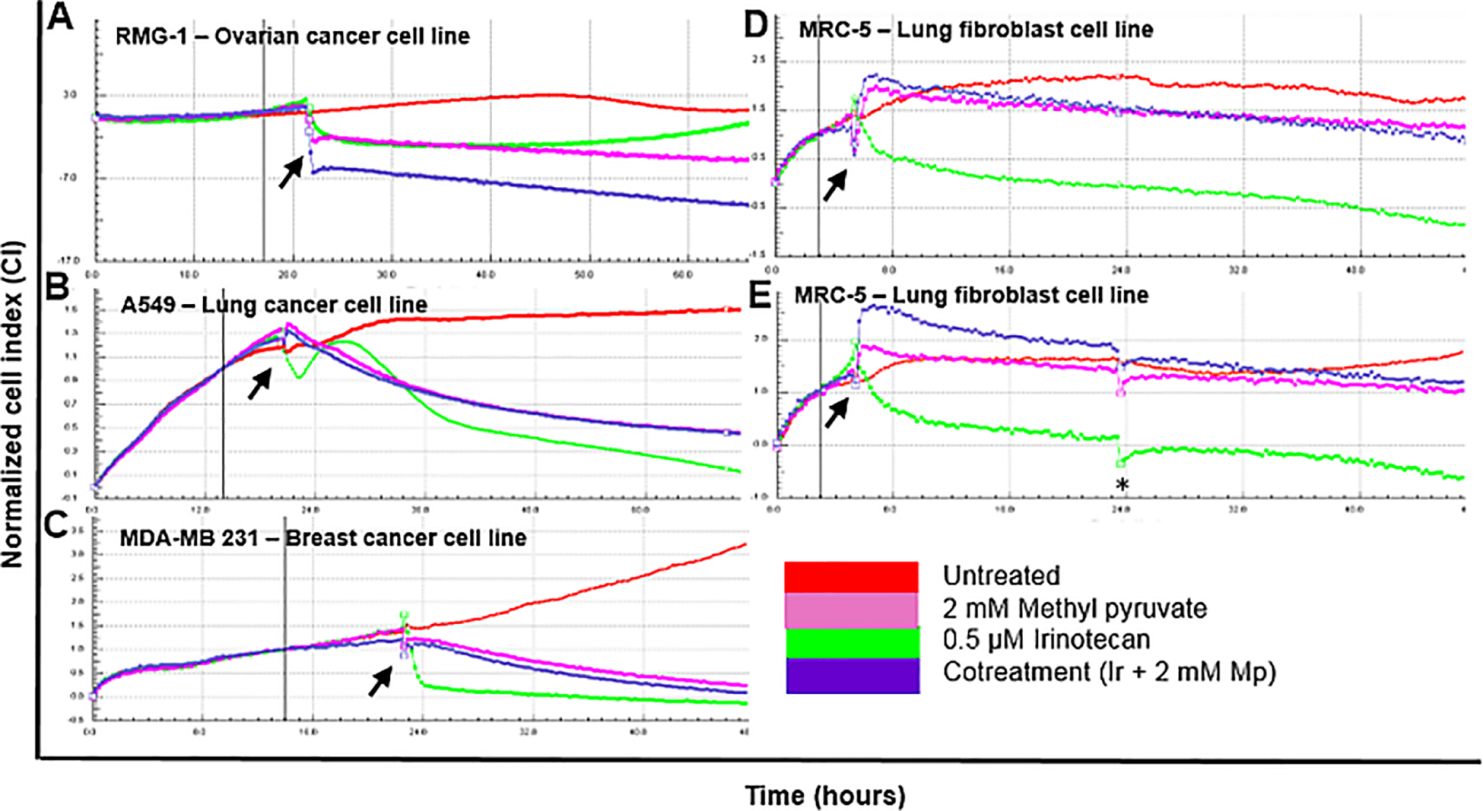
Methyl pyruvate protects normal fibroblast cell line from cell death during treatment with a chemotherapeutic agent. Three cancer cell lines including (**a**) ovarian cancer (RMG1), (**b**) lung cancer (A549), (**c**) breast cancer (MDA-MB 231 and (**d**) a normal lung fibroblast cell line (MRC-5) were seeded and grown at cell densities of 5 *x* 10^3^ for RMG-1 cells and 1 *x* 10^4^ for A549, MDA-MB 231 and MRC-5 until logarithmic phase in 16 well microelectrode E-plates. The cultures were monitored using the xCELLigence Technology. At pre-determined time points permutations of 0.5 μM irinotecan (Ir) and 2 mM methyl pyruvate (Mp) were added to the medium while the cells continued to be monitored by xCELLigence at 37°C. (**e**) After 20 hours, the medium in MRC-5 cells was replaced with fresh drug-free growth medium and the cells were grown for further 24 hours to assess the effect of methyl pyruvate on recovery from irinotecan treatment. Arrows indicate the pre-determined time points at which drugs were added. These were determined using titration curves to establish optimum cell densities. The Normalized Cell Index (NCI_ti_) was calculated as the cell index (CI_ti_) at a given time point divided by the cell index at the normalized time point (CI_nml_time_) or NCI_ti =_ CI_ti_ / CI_nml_time_). The normalized time is indicated by the vertical bold line and is for RMG-1 at 17 hours, for A549 and MDA-MB 231 cells at 14 hours and 3 hours for MRC-5 cells. The asterisk indicates replacement of growth medium in the MRC-5 cells culture. Untreated cells were used as controls. The data presented are representative of two independent experiments. A colour code is included. “cotreatment” refers to combination treatment with irinotecan and methyl pyruvate.

We hypothesised that introduction of exogenous pyruvate would interfere with the Warburg Effect and kill cancer cells. Alternatively, normal cells would benefit from this since they prefer oxidative phosphorylation to generate energy from glucose. Based on these assumptions we expected that a combination of pyruvate and irinotecan would reduce toxicity of the chemotherapeutic drug on normal cells. Irinotecan, a more water-soluble derivative of camptothecin, is often used to treat some cancers. It is used here as a DNA-damaging agent known to activate the p53 apoptotic pathway. We chose methyl pyruvate because pyruvate is unstable and has by-products that inhibit OXPHOS. Furthermore, in aqueous solutions, pyruvate is rapidly converted to parapyruvate (which inhibits the tricarboxylic acid cycle (TCA)) or to pyruvate hydrate (which is not metabolically active) [22–24]. Favorable pharmacokinetic properties such as membrane permeability further make methyl pyruvate a better substrate for the TCA compared to pyruvate [25]. As a direct input into the TCA cycle, exogenous pyruvate boosts OXPHOS, thereby interfering with the Warburg Effect. It is preferentially metabolized by mitochondrial respiration [26]. First, we used the non-invasive label-free xCELLigence RTCA (real-time cell analysis) platform on three cancer lines and on the normal lung fibroblast cell line (MRC-5) to monitor proliferation in response to treatment with methyl pyruvate, irinotecan or their combination. The xCELLigence technology uses interdigitated gold microelectrodes to monitor viability of cultured cells. Three cancer cell lines; RMG-1 (*p53*^*+/+*^), A549 (*p53*^*+/+*^),) and MDA-MB 231 (*p53*^*R280K*^) and MRC-5 were grown to exponential phase and treated as shown in (Fig 1A–1E**)**. The measure for cell growth is the Normalized Cell index (CI) as a function of time in hours.

In the cancer cell lines, the combination of methyl pyruvate and irinotecan inhibited cell proliferation independently of their p53 status (Fig 1A–1C). Indeed exogenous pyruvate alone inhibited proliferation of cancer cell lines. Importantly, methyl pyruvate reversed resistance to irinotecan that occurred in the ovarian cell line (Fig 1A). This is noteworthy because ovarian cancer, often treated with camptothecin derivatives, recurs in approximately 80% of patients necessitating alternative treatment regimens [27,28]. In contrast, methyl pyruvate protects the MRC-5 fibroblasts from irinotecan-induced cell death. In addition, pyruvate appeared to promote survival because MRC-5 cells treated with both methyl pyruvate and irinotecan survived in drug-free medium but those treated with irinotecan alone did not recover. Untreated MRC-5 fibroblasts resume exponential proliferation when allowed to recover in drug-free medium (Fig 1E). Those treated with the drug combination did not resume exponential growth during the same experimental window but did not die.

### Methyl pyruvate protects the normal fibroblast cell line by switching-off apoptotic pathways

**Fig 2:**
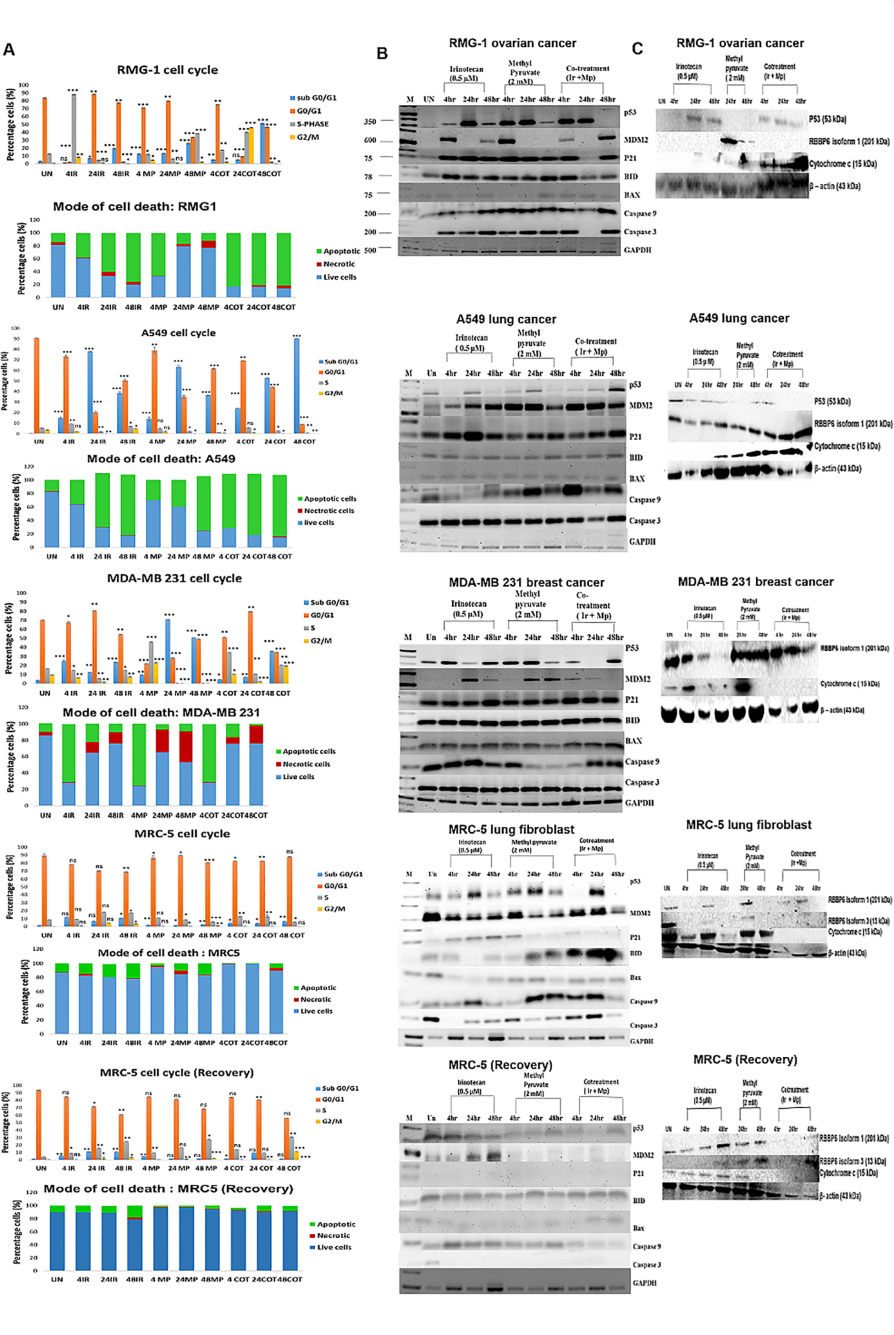
Exogenous pyruvate reduces irinotecan-induced apoptosis in normal cells while enhancing death of cancer cells. **(a)** representative FACS analyses showing cell cycle of RMG-1, A549, MDA-MB 231, and MRC-5 cell lines treated with 2 mM methyl pyruvate in the presence and absence of 0.5 μM irinotecan for 4, 24 and 48 hours respectively. **(b)** Depicts the mode of cell death for the various cell lines. Cells were stained with propidium iodide or with annexin V FITC assay. After treatment for these time periods MRC-5 cell were allowed to recover in fresh drug-free medium for a further 24 hours. The sub G_0_/G_1_ shift of cells was an indication of cell death (cells = less 2n DNA). All tests were conducted in three independent replicates. Experimental data were considered statistically significant when the p-value was ≤ 0.05 (p-value ≤ 0.001 (**) = very statistical significant, p-value ≤ 0.05 (*) = statistically significant and p-value ≥ 0.05 (ns) = not to be statistically significant). Detailed statistical data in S2 Table. Statistical analysis was done using the Graphpad software. COT: “cotreatment” indicating treatment with both irinotecan and methyl pyruvate, IR: irinotecan and Mp: methyl pyruvate. All data were acquired using the BD Accuri C6 flow cytometry.

The mechanism of cell survival and the mode of cell death were investigated by fluorescence-activated cell sorting (FACS) analysis (Fig 2A and 2B). We found that methyl pyruvate kills cancer cell lines with wild type p53 by apoptosis and the mutant p53 MDA-MB231 cells mostly by necrosis. However, only a quarter of the MDA-MB 231 (*p53*^*R280K*^) cells die by necrosis after 48 hours indicating that the efficiency of methyl pyruvate-enhanced cell death is dependent on functional p53. The reversal of irinotecan resistance shown by the ovarian cell line is again observable because irinotecan arrests the cells at G_o_/G_1_ for 48 hours in contrast with methyl pyruvate which promotes progression of the cell cycle, unlocking the irinotecan-induced G_o_/G_1_ arrest transiently, but inducing apoptosis ultimately. In sharp contrast, MRC-5 fibroblasts are protected from irinotecan-induced cell death with most cells arrested at G_o_/G_1_ during treatment and progressing to S-phase and at G_2_/M checkpoint during recovery (Fig 2A). Compared to MRC-5, the A549 cells undergo massive apoptotic death when the drugs are combined or applied individually.

The molecular underpinnings of the drug-induced phenotypes were explored by profiling gene expression using the reverse transcriptase polymerase chain reaction and Western blot analysis paying particular attention to *p53* and two of its regulators; Mouse Double Minute (MDM2) and Retinoblastoma Binding Protein 6 (RBBP6) [29,30]. p53 is often targeted by chemotherapeutic drugs and is known to be activated by irinotecan [11]. It negatively regulates glycolysis at multiple points and promotes oxidative phosphorylation by transcriptional control of the Synthesis of Cytochrome c Oxidase 2 (SCO2) gene (S1 Fig) [12,13]. Moreover, normal cells sense glucose availability by a p53-dependent pathway whereby the AMP-activated protein kinase phosphorylates p53 on serine 15 resulting in cell cycle arrest at G_1_/S [16]. Presumably, *Mdm2* and RBBP6, which are known p53 negative regulators [29,30], are also involved in glucose metabolism.

**Fig 3:**
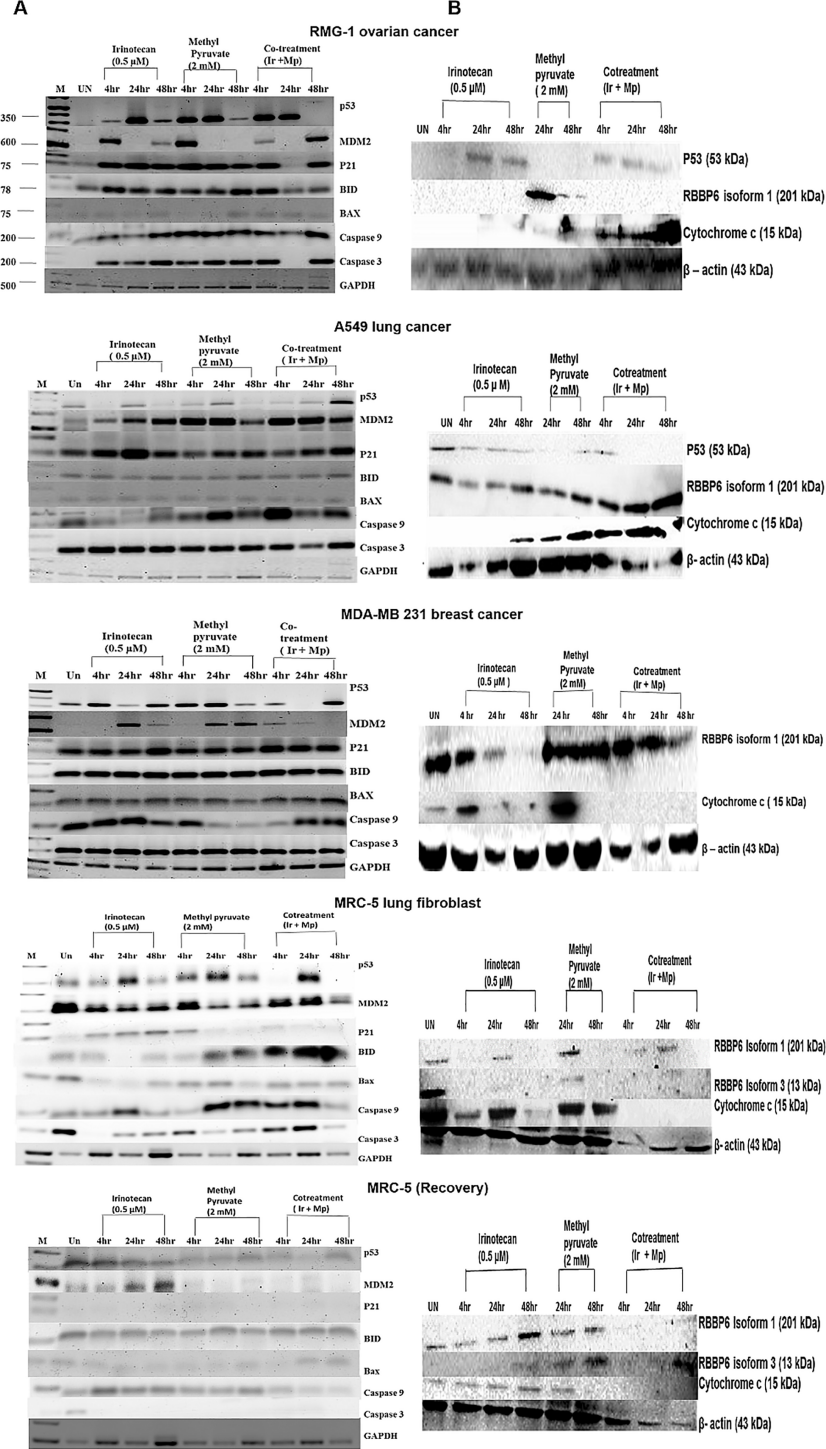
Analysis of apoptotic pathways. **(a)** RT-PCR shows changes in transcription of p53, MDM2, p21, BID, Bax, caspase 9, and caspase 3 after 4 hours, 24 hours and 48 hours treatment with 2 mM methyl pyruvate in the presence and absence 0.5 μM irinotecan in RMG-1, A549, MDA-MB 231, and MRC-5 cells including MRC-5 cells during the recovery period. Glyceraldehyde 3-phosphate dehydrogenase (GAPDH) was used as a loading control. Semi-quantitative analysis in S2 Fig. **(b)** Western Blot analysis showing changes in expression of p53, RBBP6 and cytochrome c proteins in RMG1, A549, MDA-MB 231, and MRC5 cell lines after treatment as indicated. Again MRC5 cells were allowed to recovery for a further 24 hours after each treatment. Actin was used a loading control. Densitometry semi-quantitative analysis shown in S3 Fig.

To elucidate the mechanism by which methyl pyruvate antagonizes apoptotic pathways we explored the expression profiles of pro-apoptotic and anti-apoptotic genes in response to treatment by various combinations of the drugs. Except for the ovarian and MRC-5 cell lines, combination of methyl pyruvate and irinotecan enhanced transcription of p53 after 48 hours in cancer cell lines (Fig 3A). In MRC-5 cells and in contrast with the cancer cell lines, it is notable that p21 transcription is abrogated when methyl pyruvate is introduced. Furthermore, pro-apoptotic gene transcription, including, *Bax*, *caspase 9* and *caspase 3*, is also abrogated in MRC-5 cells in comparison with all the cancer cell lines. The crucial cytochrome c, which activates the effector caspase-3, was highly expressed in cancer cell lines treated with the drug combination but not in the MRC-5 cells and in the mutant p53 MDA-MB231 cells which die by necrosis. In MDA-MB231 cells, *Mdm2* is also not affected by changes in p53 expression consistent with the fact that the mutation is in the DNA-binding domain of the protein. Interestingly, there is transient depletion of *p21* and *caspase 3* transcripts between 4–24 hours in RMG-1 cells treated with both drugs. This coincides with the burst of cell cycle progression into the S- and G_2_M phases detected by FACS (Fig 2). Cell death and arrest at G_o_/G_1_ returns in 48 hours accompanied by reappearance of p21 and caspase 3. It is noteworthy that when *p21* reappears, depletion of p53 and increase in *Mdm2* mRNA is observed, suggesting deployment of the p53/MDM2 feedback mechanism. In addition, Western blot analysis, shows persistence, albeit at reduced levels, of the p53 protein and depletion of RBBP6 (Fig 3B) suggesting that there may be a tight control of this axis in the ovarian cell line as the opposite protein profile is observed in the other cancer cell lines. Thus, in contrast with the cancer cell lines, the combination of methyl pyruvate and irinotecan attenuates both the p53/p21-mediated and mitochondrial apoptosis in MRC-5 fibroblasts during treatment and appears to deplete both pathways beyond detection during recovery. This is demonstrated strongly by the undetectable cytochrome c and effector caspases in MRC-5 fibroblasts and suggests that apoptosome formation does not occur. Furthermore, in MRC-5 fibroblasts, these drugs activate differential expression of two isoforms of RBBP6. Isoform 1 is expressed in the first 24 hours of treatment with the drug combination and depleted during recovery. On the other hand, isoform 3 is upregulated within 24 hours of the recovery period.

### Methyl pyruvate promotes cell death in cancer cell lines but survival in the normal lung fibroblast cell line

**Fig 4:**
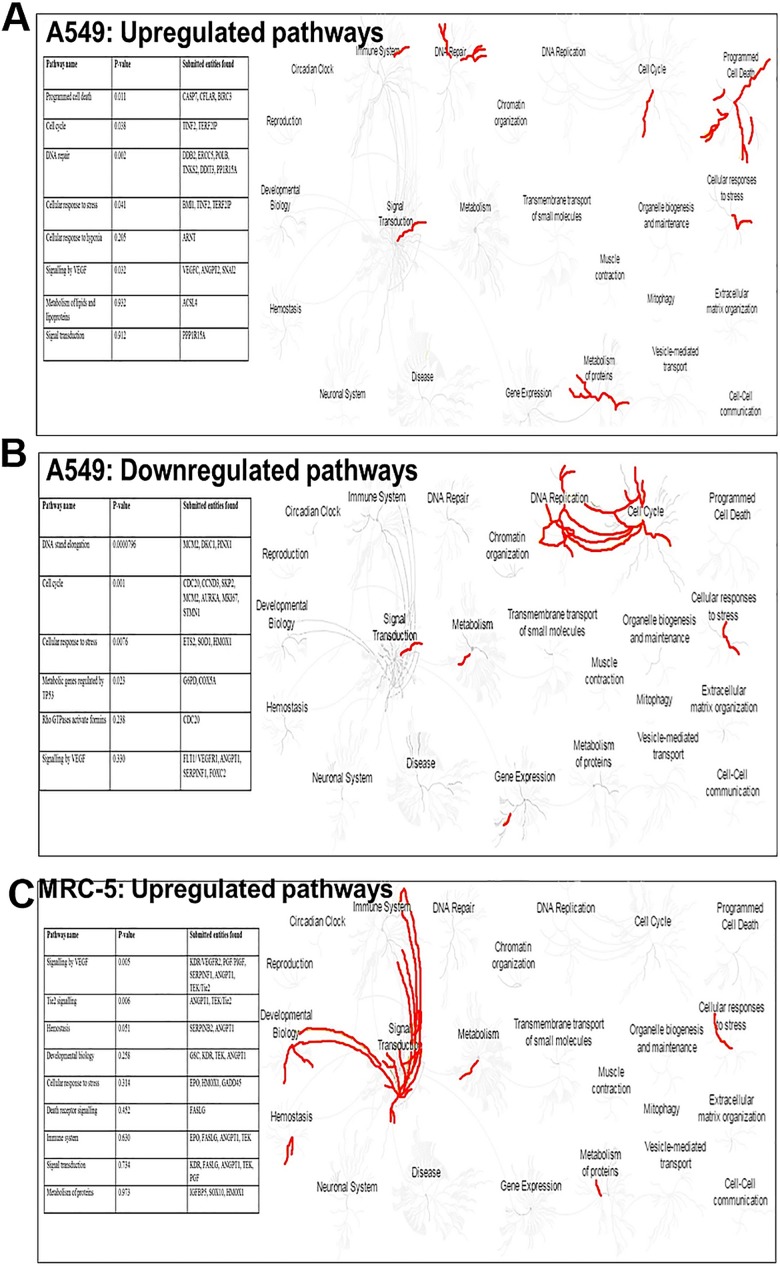
Methyl pyruvate upregulates pro-survival and anti-apoptotic genes in MRC-5 fibroblasts and contrasting genes in A549 cancer cells. The RT^2^ Profiler^TM^ PCR array Human Cancer PathwayFinder^TM^ was used to analyse differential expression of 84 cancer-related genes in response to treatment with methyl pyruvate and irinotecan for 48 hours. Gene expression in treated cells was compare with expression in corresponding cells, thus four experiments were conducted. **(a)** The Venn diagram summarizes differentially expressed genes in A549 and MRC-5 cells. **(b)** Using PANTHER the differentially expressed genes were further analysed to reveal biological pathways that were affected when the A549 and MRC-5 cells are treated with irinotecan and methyl pyruvate. Uniquely expressed genes were used as input into PANTHER to identify perturbed biological pathways. The Hidden Markov statistical model (HMM) was used to assign genes from the query list to their corresponding biological pathways. **(c)** The GeneMANIA module in Cytoscape (version 3.3.0) was applied to construct interaction networks in each cell line. The Networks represent upregulated and downregulated gene sets after treatment with both drugs for 48 hours. Genes in the submitted query list are indicated as circular nodes while those predicted to be related are displayed as diamond nodes (<>). Pathway categories received a score weight when the pathways data sets in GeneMANIA link to members of the query list. The edges (coloured lines) that connect neighbouring genes are depicted as follows: **medium purple**: Co-expression, **medium turquoise**: pathway; **brown**: physical interaction; **blue**: co-localization; **khaki**:shared protein domains; **maroon**: genetic interactions; **green**: predicted. Interactions were considered statistically significant when q < 0.05. A q-value of 0.05 indicates that there is a 5% chance of getting a false statistically significant result. The q-values were estimated by the in-built Benjamini-Hochberg procedure.

To investigate molecular pathways affected by the drug combination on normal and cancer cells, we conducted a focused pathway study using the Human Cancer PathwayFinder^TM^ RT^2^ Profiler^TM^ PCR Array, (PAHS-O33ZF-12) which profiles 84 cancer-related genes. The data were analysed by QIAGEN data analysis software, PANTHER (**P**rotein **An**alysis **T**hrough **E**volutionary **R**elationships), Genemania Cytoscape and REACTOME. Using QIAGEN data analysis software, 13 and 17 genes were found to be uniquely upregulated in MRC-5 cells and in A549 cells respectively (Fig 4A). Using PANTHER and Genemania, the results indicate that most upregulated genes in A549 cells mediate apoptosis (Fig 4B). In contrast, those upregulated in the MRC-5 lung fibroblasts promote angiogenesis, cell cycle regulation, cell survival and control glucose metabolism. The drug-induced pro-angiogenic pathways in MRC-5 fibroblasts are mediated by known pro-angiogenic factors and by molecules classified as components of blood coagulation and plasminogen activating cascades. These include erythropoietin/thrombopoietin (EPO), Growth Arrest DNA-damage-inducible protein 45 (GADD45G), FASL (Fas ligand, also known as *TNFSF6/CD95L*)), Goosecoid (GSC), SRY-box 10 (SOX10), KDR (a VEGFR-2 receptor), TEK/Tie2 (Tyrosine kinase endothelial receptor), IGFBP5 (Insulin like growth factor binding protein 5), SERPINFI (Serpin peptidase inhibitor clade F1, member 1), Angiopoietin-1 (ANGPT1), placenta growth factor (PGF), Heme_oxygenase (HMOX1) and SERPINB2 (Serpin peptidase inhibitor, clade B, member 2) (S3 Table). Cytoscape, which predicts gene networks using a ‘guilt-by-association’ approach, shows that pro-angiogenic proteins, notably ANGPT1 are downregulated in A549 cells and anti-angiogenic proteins, including ANGPT2 are upregulated. Also notable is the downregulation of mitotic gene networks (Fig 4C). The opposite effect is observed in similarly treated MRC-5 fibroblasts where upregulated networks mediate cell survival.

**Fig 5:**
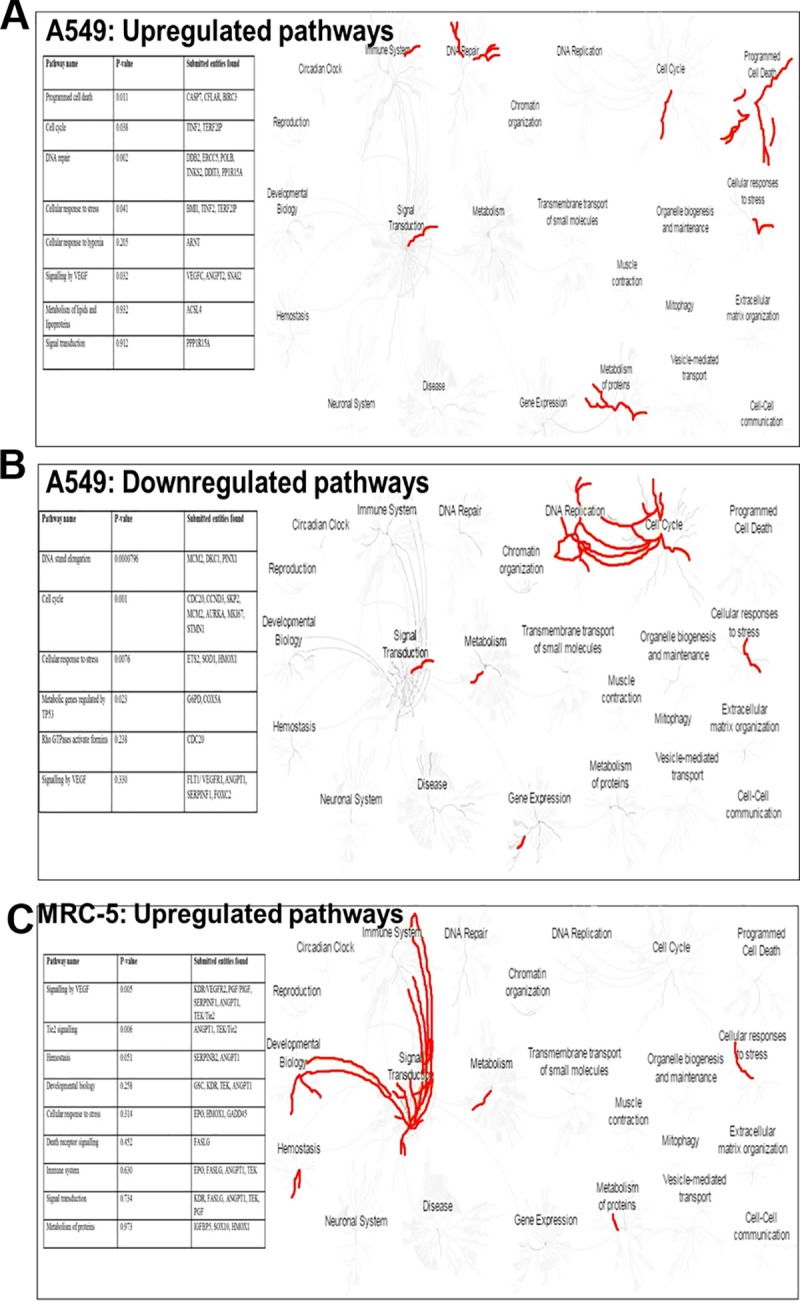
Pathway analysis. In the cancer fibroblasts the irinotecan-methyl pyruvate combination cause disruption of major signaling pathways and cellular processes and upregulates angiogenesis and cell cycle progression in the MCR-5 fibroblasts. REACTOME analyses was conducted using differentially query lists of differentially expressed gene genes in A549 and MRC-5 cells. Pathways were considered enriched when a significant number of the query list genes were part of a particular pathway against the overall pathway genes. Each pathway was considered statistically enriched when the p < 0.05. REACTOME produced maps of 24 pathways highlighted (in red) induced and downregulated ones in (**a-b**) A549 and (**c**) MRC-5 cells. **Insert tables**: list upregulated and downregulated genes. Each pathway was considered statistically enriched when the p < 0.05. In-depth analyses of the pathways are presented in S4 Fig.

REACTOME analysis shows that the methyl pyruvate and irinotecan combination upregulates apoptotic pathways, mobilizes DNA repair pathways, inhibits VEGF signaling and downregulates cell cycle checkpoint genes in A549 cells Fig 5A and 5B (**S4 Fig** On the other hand, in MRC-5 fibroblasts, VEGF signaling is activated and apoptotic pathways are not elevated (Fig 5C). In-depth REACTOME analysis reveals that in A549 cells, major disruption of cellular processes occurs including, collapse of the replication fork, disruption of the pentose phosphate pathway (downregulation of Glucose-6-phosphate dehydrogenase), downregulation of metabolic genes regulated by TP53, telomere stress-induced senescence and necrosis **S4 Fig**. The REACTOME analysis also points to ETS1 and ETS2 transcription factors being downregulated in the oxidative stress induced senescence pathway S4 Fig. This suggests a decline in MEK signaling which is mediated by ETS transcription factors in replicative senescence [31]. The MEK/ERK signaling cascade is well-known to play a role in regulation of various cellular processes including, proliferation, differentiation, survival, and development.

## Discussion

In this study, we used the xCELLigence technology to show that methyl pyruvate protects a normal lung fibroblast cell line from irinotecan-induced cell death but fails to do so for cancer cell lines, especially for the comparable lung cancer cell line. We also elucidate the molecular underpinnings of this phenomenon and propose molecular pathways that may be involved in the protection mechanism. Our findings, based on the use of different cancer cell lines, indicate that the impact of methyl pyruvate depends upon cellular contexts and may be influenced by p53. In a previous study, using wild type *Drosophila*, we showed that methyl pyruvate reduces the toxic effects of camptothecin, a DNA damaging agent of which irinotecan is derived from [32]. Interestingly, our results reinforce previous findings by other researchers. Recently, another pyruvate ester, ethyl pyruvate was found to selectively kill leukemia cells [33]. Another study shows that ethyl pyruvate increased survival of mice undergoing radiation treatment [34]. Because, these observations have important implications for managing toxicity during chemotherapy, we shall discuss the molecular pathways involved in the protective mechanisms offered by the introduction of exogenous pyruvate during treatment with a chemotherapeutic agent such as irinotecan.

### Exogenous pyruvate deregulates apoptotic pathways

Our findings indicate that methyl pyruvate protects MRC-5 fibroblasts from irinotecan-induced cell death by turning off the p53/p21 axis since the downstream p21 is downregulated upon treatment by the drug combination and undetectable during the recovery period. The importance of p21 is underlined by the unlocking of the cell cycle arrest at G_o_/G_1_ when p21 is absent in the ovarian cell line. This phenotype is rescued when p21 is expressed by the cells at a later stage. The intrinsic apoptotic pathway appears to be shut down remarkably during the recovery period as many of the key pro-apoptotic molecules investigated were not detected by RT-PCR. Irinotecan alone induces the p53/p21 axis on the lung fibroblast cancer cell line and the MRC-5 fibroblasts but when exogenous pyruvate is added both pathways are abrogated in the normal cell line while activated in eth cancer cell lines. We also investigated a possible role for negative regulators of p53; MDM2 and RBBP6 because the former is a target of nutlins while the latter is a potential drug target. We found that two isoforms of RBBP6 are inducible by these drug treatments in cancer and normal cells. Isoform 1 was induced in the cancer cell lines while isoform 1 and 3 were differentially expressed in the MRC-5 fibroblast. RBBP6 probably influences the p53/p21 apoptotic axis by employing a self-regulatory mechanism whereby isoform 3 acts as a competitive inhibitor of the larger isoforms [35]. Isoform 1 is a negative regulator of p53 using its E3 ligase activity to promote p53 degradation, and has anti-apoptotic properties whereas the DWNN module (isoform 3), that are characterized by the ubiquitin-like Domain With No Name (DWNN) has pro-apoptotic influence. Indeed, Isoform 3 is depleted in some cancers [36]. This regulatory mechanism probably occurs in the S- and G_2_/M-cell cycle phases where methyl pyruvate facilitates cell cycle progression (Fig 2A). These stages are tightly controlled by p53 and pRb, both of which bind to RBBP6 [30,37].

### Exogenous pyruvate promotes cell survival in the normal fibroblast cell line and cell death in cancer cell lines

Using the focused Human Cancer PathwayFinder^TM^ Array (PAHS-O33ZF-12) we analysed differential expression of genes in the MRC-5 cells and in A549 cells. Most of the genes that were upregulated in MRC-5 fibroblasts support biological processes that benefit cell survival. Generally, these proteins keep balanced homeostasis and promote cell differentiation and survival. And a molecule like Goosecoid is known to function in glucose metabolism by targeting genes that are active in the liver, pancreas, and adipose tissue [38]. GADD45G might be responsible for cell cycle progression observed in Fig 2A because it specifically interacts with the Cdk1/CyclinB1 complex and aids in controlling the S and G_2_/M checkpoints [39,40]. While ANGPT1 is upregulated in the MRC-5 fibroblasts, the antagonistic ANGPT2 is upregulated in A549 cells suggesting that methyl pyruvate directly triggers opposite outcomes in these cells: pro-angiogenic and pro-survival factors in the MRC-5 fibroblasts and anti-angiogenic factors in A549 cells.

Moreover, It appears that generally methyl pyruvate alone induces apoptosis in cancer cells including in the resistant RMG-1 (ovarian) cells and does the opposite in MRC-5 fibroblasts. Irinotecan is known to induce apoptosis by activating p53 and Bcl-2 family proteins and to induce G_2_/M arrest and apoptosis in colon and testicular cancers [41,42]. Consequently, irinotecan should affect p21^Waf1/Cip1/Sdi1^, the major effector of cell cycle arrest after DNA damage and key cyclin-dependent kinase inhibitor that is induced by p53-dependent and p53-independent ways. Indeed, p21 overexpression leads to G_1_, G_2_ or S-phase arrest by inhibiting a subset of genes involved in mitosis, DNA replication, segregation, and repair [43]. In the current study, we report that a transient p21 depletion observed in RMG-1 cells is accompanied by cell cycle progression. Furthermore, in MRC-5 fibroblasts, exogenous pyruvate abrogates irinotecan-induced p21 transcription whether introduced on its own or with irinotecan.

## Conclusion

This study shows that exogenous pyruvate protects irinotecan-treated normal lung fibroblast cell line and induces apoptosis in a lung cancer cell line. We propose that methyl pyruvate has potential to protect normal cells during chemotherapy.

## Supporting information

S1 Fig p53 impact on Glucose metabolism. Under physiological conditions, the tumour suppressor protein p53 controls glycolysis and the oxidative phosphorylation. p53 potentially influences glycolysis at multiple points starting with cellular intake via the glucose transport proteins (GLUT1-4) and then at downstream points involved in breakdown of glucose to pyruvate. Furthermore, p53 promotes oxidative phosphorylation by transactivation of Synthesis of Cytochrome c Oxidase 2 (SCO2). Broadly, p53 negatively regulates glycolysis while promoting oxidative phosphorylation and the pentose phosphate pathway.
(TIF)

S2 Fig Exogenous pyruvate reduces irinotecan-induced apoptosis in normal cells while enhancing death of cancer cells. (A) representative FACS analyses showing cell cycle of RMG-1, A549, MDA-MB 231, and MRC-5 cell lines treated with 2 mM methyl pyruvate in the presence and absence of 0.5 μM irinotecan for 4, 24 and 48 hours respectively. Total DNA was quantitatively measured by staining with propidium iodide. (B) Analyses of flow cytometry sub G0/G1 cell distribution in RMG-1, A549, MDA-MB 231, MRC-5, and MRC-5 upon recovery. The sub G0/G1 shift of cells was an indication of cell death since this phase of the cell cycle is characterized by cells consisting of less than 2n DNA. All tests were conducted in three independent replicates.
(TIF)

S3 Fig **Relative transcript (A) and protein (B) quantification normalized by the expression of the house keeping genes,** GAPDH and β-actin using MyImage AnalysisTM Software (Thermo Scientific) from one independent experiment in RMG-1 ovarian cancer cell line, A549 lung cancer cell line, MDA-MB 231 breast cancer cell line, MRC5 a non-tumorigenic cell line and MRC5 cells upon recovery from various treatments.
(TIF)

S4 Fig Pathway analysis using REACTOME. Pathway diagrams were constructed using the REACTOME pathway analysis software. Pathway diagrams are a representation of steps or processes of pathways with interconnected molecular events. Unique genes with altered expression patterns between A549 and MRC-5 cells were submitted as the query list onto the REACTOME web portal. Pathways were enriched when a significant number of the query list genes were part of a particular pathway against the overall pathway genes. Each pathway was considered statistically enriched when the p < 0.05. The dark green colour represents genes with upregulated expression levels while the bright yellow colour represents downregulated genes in a step or process. In A549 lung cancer cells, the combined treatment upregulated genes involved in (A); regulation of necrosis (p = 0.56E-5), intrinsic programmed cell death (p = 2.22E-2), packaging of telomere ends (p = 1.9E-2), dual inclusion GC:NER (p = 2 E-3), recruitment of POLB to AP site: abasic sugar-phosphate removal (p = 1.44E-2), cellular response to hypoxia (p = 1.19E-1), signaling by VEGF (p = 6.26E-1), and telomere stress induced senescence (p = 4.46E-2). Furthermore, in A549 lung cancer cells, the combined treatment downregulated genes involved in (B); DNA strand elongation: unwinding of DNA (p = 7.53E-6), activation of pre-replicative complex (p = 6.66E-5), mitotic G0/G1/S phase (p = 6.21E-4), signaling by VEGF (p = 3.31E-1), cellular response to oxidative stress (p = 5.86E-4), detoxification of ROS (p = 1.44E-3), and metabolic genes regulated by TP53 (p = 2.35E-2). In MRC-5 normal lung fibroblast cells, the combined treatment upregulated genes involved in (C); signaling by VEGF (p = 3.02E-4), Tie2 signaling (p = 3.38E-2), regulation and transport of IGF by IGFBP5 (p = 3.61E-2), Dissolution of fibrin clot (fibrinolysis) (p = 3.42E-2),cellular response to hypoxia (p = 1.32E-2), POU5F1 (OCT4), S0x2, NANOG repress genes related to differentiation (p = 1.72E-2), and heme degradation (p = 2.02E-2). The REACTOME key diagram below gives detail description of the icons used.
(TIF)

S1 Table Primer sets of each gene to be amplified. (PDF)

S2 Table Detailed statistical data. (PDF)

S3 Table Information of differentially expressed genes. **(**A) 13 upregulated MRC-5 genes (B) 17 upregulated A549 genes (C) 18 upregulated A549 genes.
(PDF)
