# Correction: Methyl pyruvate protects a normal lung fibroblast cell line from irinotecan-induced cell death: Potential use as adjunctive to chemotherapy

**DOI:** 10.1371/journal.pone.0194260

**Published:** 2018-04-09

**Authors:** The PLOS ONE Staff

There is an error in Fig 1. The axes are not individually labeled. Please find the correct Fig 1 here.

**Fig 1:**
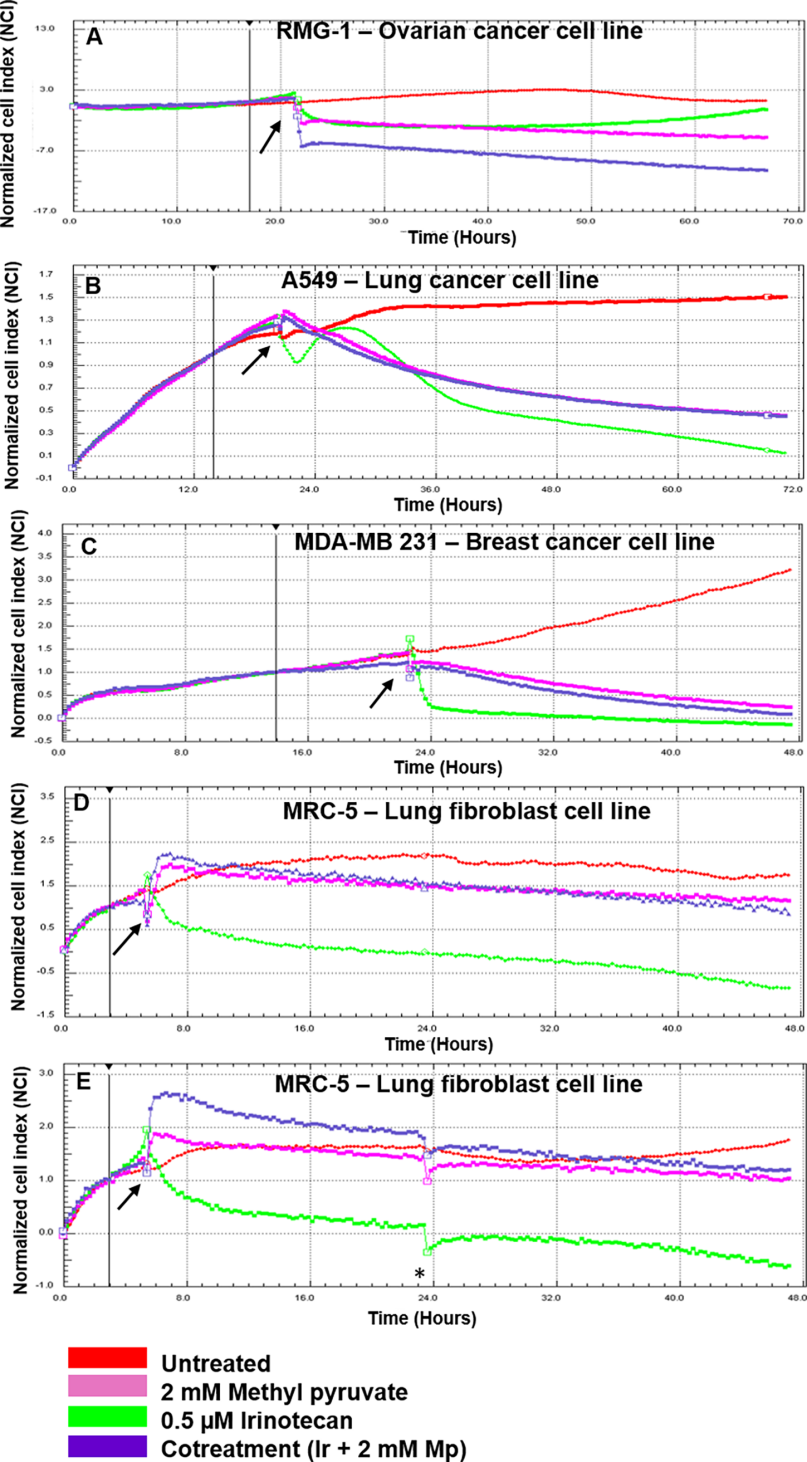
Methyl pyruvate protects normal fibroblast cell line from cell death during treatment with a chemotherapeutic agent. Three cancer cell lines including (**a**) ovarian cancer (RMG1), (**b**) lung cancer (A549), (**c**) breast cancer (MDA-MB 231 and (**d**) a normal lung fibroblast cell line (MRC-5) were seeded and grown at cell densities of 5 x 10^3^ for RMG-1 cells and 1 x 10^4^ for A549, MDA-MB 231 and MRC-5 until logarithmic phase in 16 well microelectrode E-plates. The cultures were monitored using the xCELLigence Technology. At pre-determined time points permutations of 0.5 μM irinotecan (Ir) and 2 mM methyl pyruvate (Mp) were added to the medium while the cells continued to be monitored by xCELLigence at 37°C. (**e**) After 20 hours, the medium in MRC-5 cells was replaced with fresh drug-free growth medium and the cells were grown for further 24 hours to assess the effect of methyl pyruvate on recovery from irinotecan treatment. Arrows indicate the pre-determined time points at which drugs were added. These were determined using titration curves to establish optimum cell densities. The Normalized Cell Index (NCI_ti_) was calculated as the cell index (CI_ti_) at a given time point divided by the cell index at the normalized time point (CI_nml_time_) or NCI_ti_ = CI_ti_ / CI_nml_time_). The normalized time is indicated by the vertical bold line and is for RMG-1 at 17 hours, for A549 and MDA-MB 231 cells at 14 hours and 3 hours for MRC-5 cells. The asterisk indicates replacement of growth medium in the MRC-5 cells culture. Untreated cells were used as controls. The data presented are representative of two independent experiments. A colour code is included. “cotreatment” refers to combination treatment with irinotecan and methyl pyruvate.

The images in Fig 3 are incorrectly duplicated in Fig 2B and Fig 2C. Please see the correct Fig 2 here.

**Fig 2:**
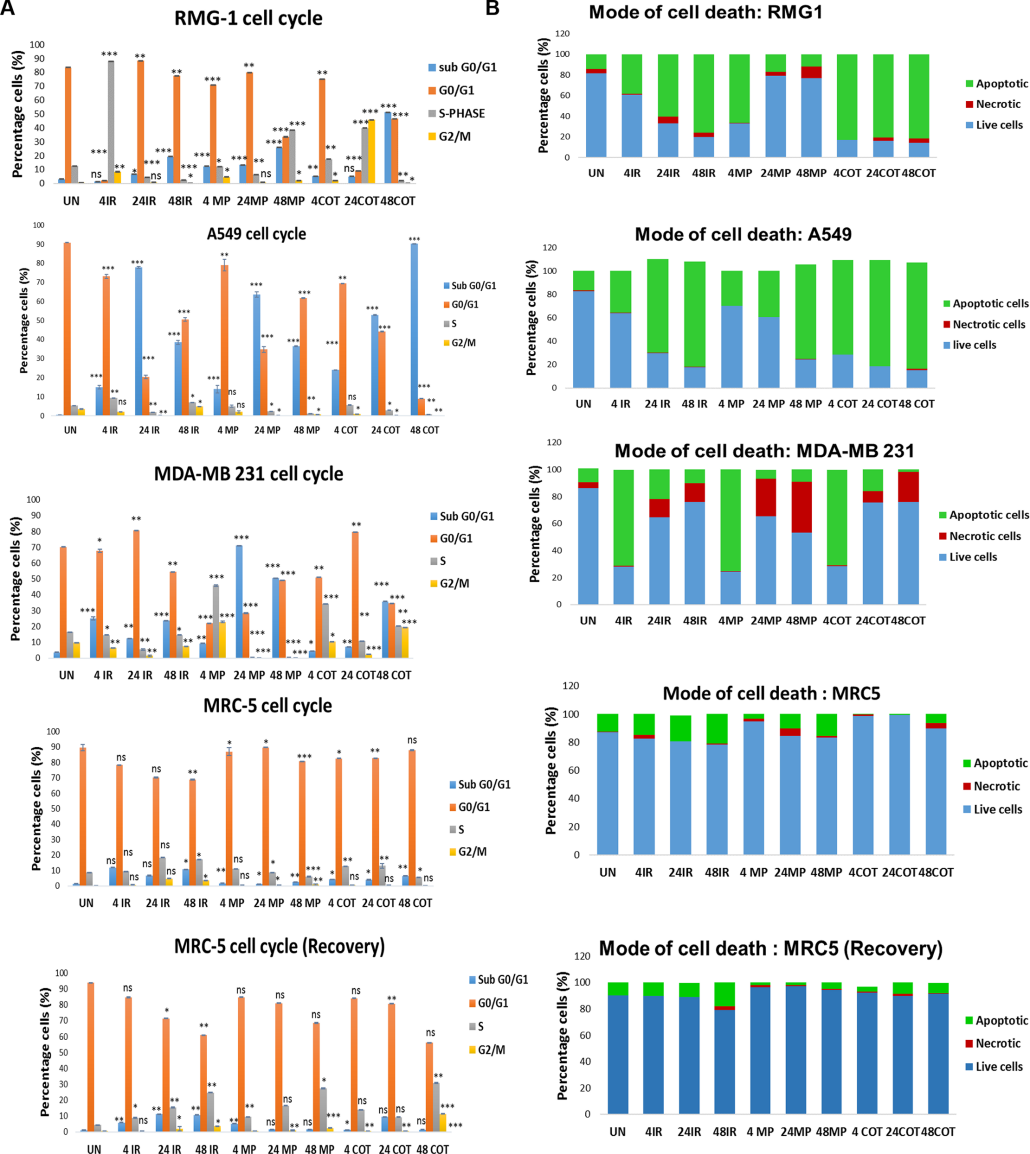
Exogenous pyruvate reduces irinotecan-induced apoptosis in normal cells while enhancing death of cancer cells. **(a)** representative FACS analyses showing cell cycle of RMG-1, A549, MDA-MB 231, and MRC-5 cell lines treated with 2 mM methyl pyruvate in the presence and absence of 0.5 μM irinotecan for 4, 24 and 48 hours respectively. **(b)** Depicts the mode of cell death for the various cell lines. Cells were stained with propidium iodide or with annexin V FITC assay. After treatment for these time periods MRC-5 cell were allowed to recover in fresh drug-free medium for a further 24 hours. The sub G_0_/G_1_ shift of cells was an indication of cell death (cells = less 2n DNA). All tests were conducted in three independent replicates. Experimental data were considered statistically significant when the p-value was ≤ 0.05 (p-value ≤ 0.001 (**) = very statistical significant, p-value ≤ 0.05 (*) = statistically significant and p-value ≥ 0.05 (ns) = not to be statistically significant). Detailed statistical data in S2 Table. Statistical analysis was done using the Graphpad software. COT: “cotreatment” indicating treatment with both irinotecan and methyl pyruvate, IR: irinotecan and Mp: methyl pyruvate. All data were acquired using the BD Accuri C6 flow cytometry.

Fig 5 is incorrectly duplicated in Fig 4. Please see the correct Fig 4 here.

**Fig 4:**
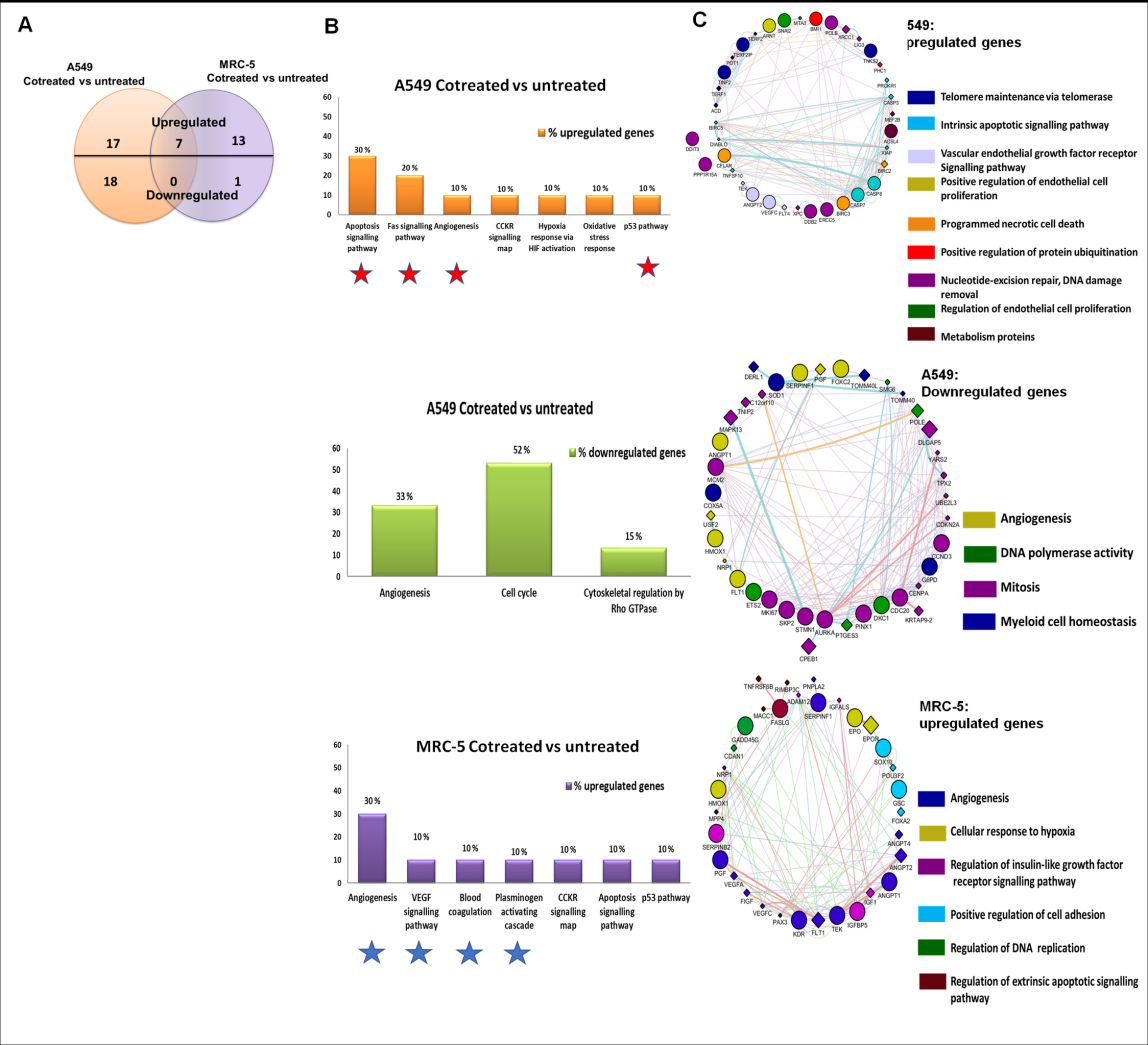
Methyl pyruvate upregulates pro-survival and anti-apoptotic genes in MRC-5 fibroblasts and contrasting genes in A549 cancer cells. The RT^2^ Profiler^TM^ PCR array Human Cancer PathwayFinder^TM^ was used to analyse differential expression of 84 cancer-related genes in response to treatment with methyl pyruvate and irinotecan for 48 hours. Gene expression in treated cells was compare with expression in corresponding cells, thus four experiments were conducted. **(a)** The Venn diagram summarizes differentially expressed genes in A549 and MRC-5 cells. **(b)** Using PANTHER the differentially expressed genes were further analysed to reveal biological pathways that were affected when the A549 and MRC-5 cells are treated with irinotecan and methyl pyruvate. Uniquely expressed genes were used as input into PANTHER to identify perturbed biological pathways. The Hidden Markov statistical model (HMM) was used to assign genes from the query list to their corresponding biological pathways. **(c)** The GeneMANIA module in Cytoscape (version 3.3.0) was applied to construct interaction networks in each cell line. The Networks represent upregulated and downregulated gene sets after treatment with both drugs for 48 hours. Genes in the submitted query list are indicated as circular nodes while those predicted to be related are displayed as diamond nodes (<>). Pathway categories received a score weight when the pathways data sets in GeneMANIA link to members of the query list. The edges (coloured lines) that connect neighbouring genes are depicted as follows: **medium purple**: Co-expression, **medium turquoise**: pathway; **brown**: physical interaction; **blue**: co-localization; **khaki**:shared protein domains; **maroon**: genetic interactions; **green**: predicted. Interactions were considered statistically significant when q < 0.05. A q-value of 0.05 indicates that there is a 5% chance of getting a false statistically significant result. The q-values were estimated by the in-built Benjamini-Hochberg procedure.

The publisher apologizes for these errors.
