# Supplementary figures and images for: Methyl pyruvate protects a normal lung fibroblast cell line from irinotecan-induced cell death: Potential use as adjunctive to chemotherapy
Source: PLoS One. 2017 Aug 10;12(8):e0182789. doi: 10.1371/journal.pone.0182789 (PMC5552298; doi:10.1371/journal.pone.0182789)

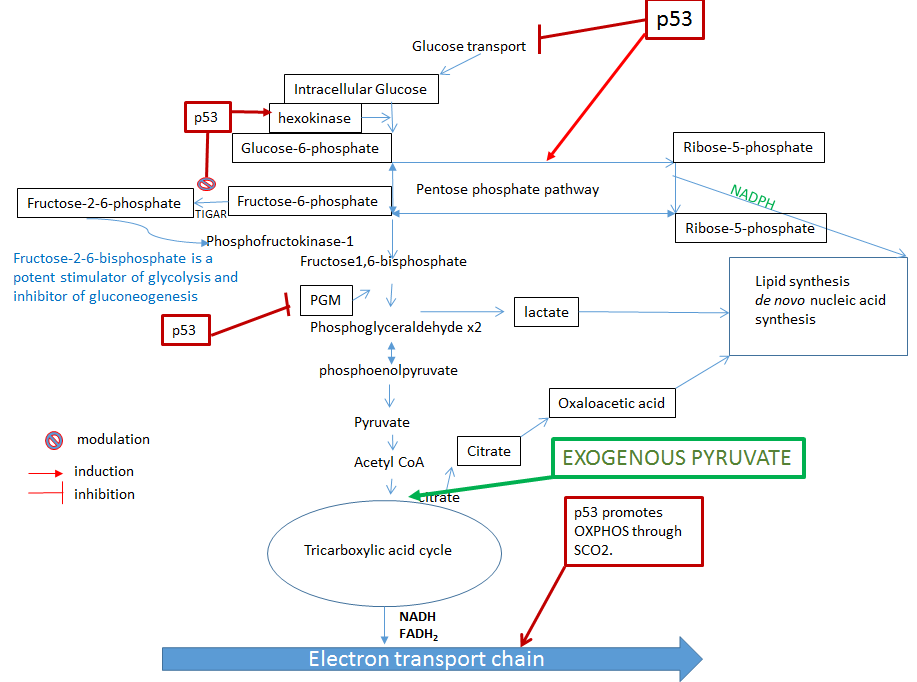

Supplement: S1 Fig — Under physiological conditions, the tumour suppressor protein p53 controls glycolysis and the oxidative phosphorylation. p53 potentially influences glycolysis at multiple points starting with cellular intake via the glucose transport proteins (GLUT1-4) and then at downstream points involved in breakdown of glucose to pyruvate. Furthermore, p53 promotes oxidative phosphorylation by transactivation of Synthesis of Cytochrome c Oxidase 2 (SCO2). Broadly, p53 negatively regulates glycolysis while promoting oxidative phosphorylation and the pentose phosphate pathway. (TIF) [file pone.0182789.s001.tif]

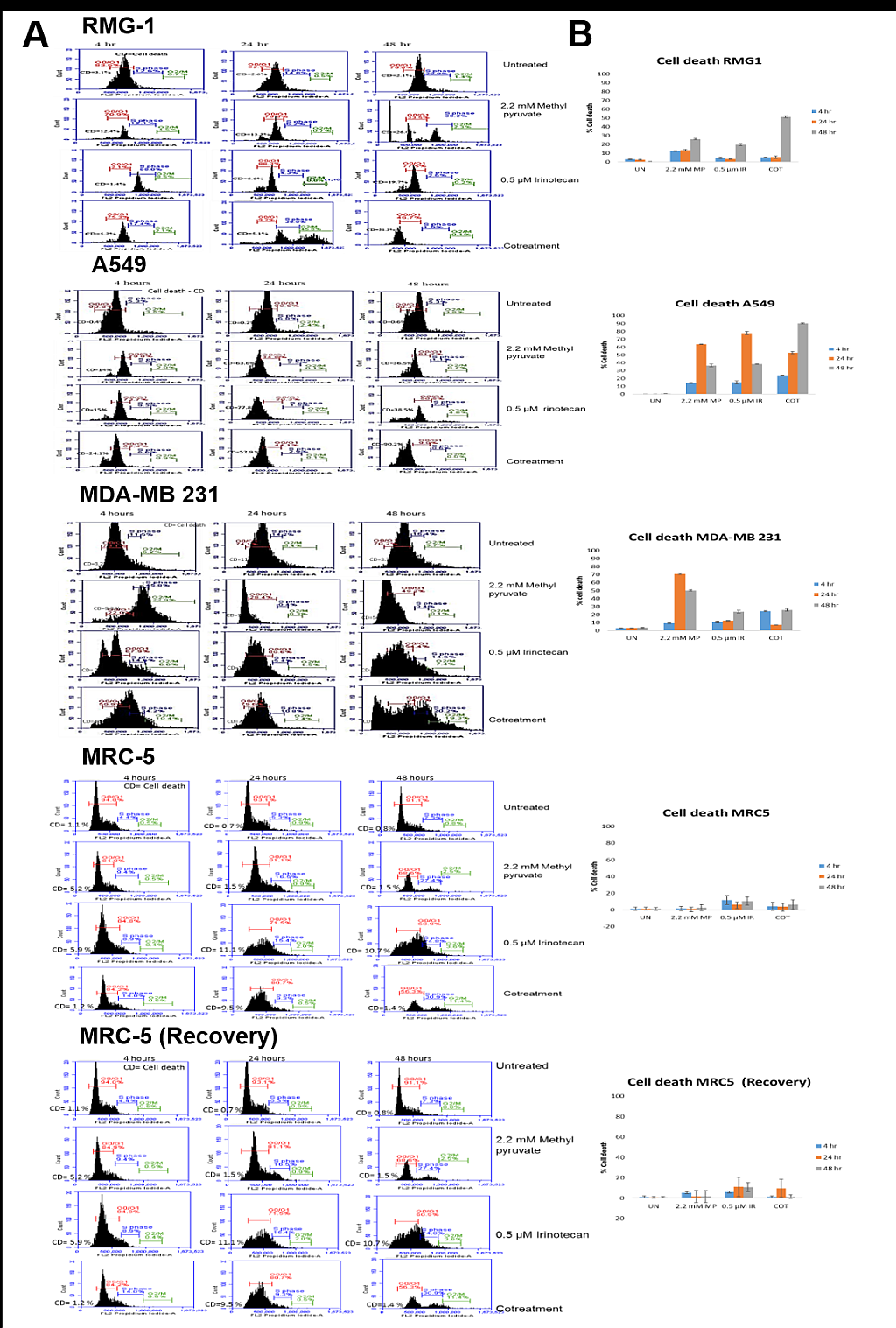

Supplement: S2 Fig — (A) representative FACS analyses showing cell cycle of RMG-1, A549, MDA-MB 231, and MRC-5 cell lines treated with 2 mM methyl pyruvate in the presence and absence of 0.5 μM irinotecan for 4, 24 and 48 hours respectively. Total DNA was quantitatively measured by staining with propidium iodide. (B) Analyses of flow cytometry sub G0/G1 cell distribution in RMG-1, A549, MDA-MB 231, MRC-5, and MRC-5 upon recovery. The sub G0/G1 shift of cells was an indication of cell death since this phase of the cell cycle is characterized by cells consisting of less than 2n DNA. All tests were conducted in three independent replicates. (TIF) [file pone.0182789.s002.tif]

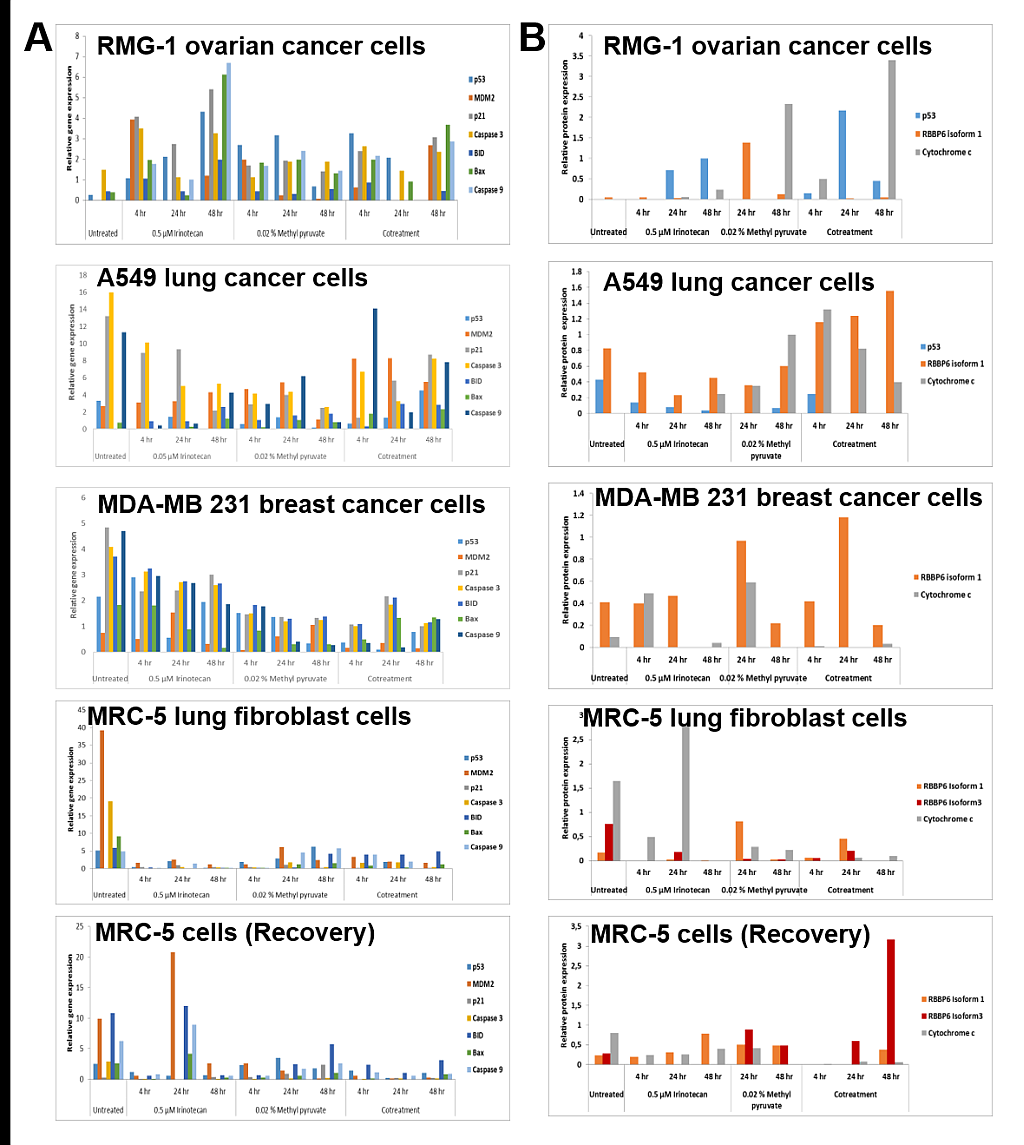

Supplement: S3 Fig — Relative transcript (A) and protein (B) quantification normalized by the expression of the house keeping genes, GAPDH and β-actin using MyImage AnalysisTM Software (Thermo Scientific) from one independent experiment in RMG-1 ovarian cancer cell line, A549 lung cancer cell line, MDA-MB 231 breast cancer cell line, MRC5 a non-tumorigenic cell line and MRC5 cells upon recovery from various treatments. (TIF) [file pone.0182789.s003.tif]

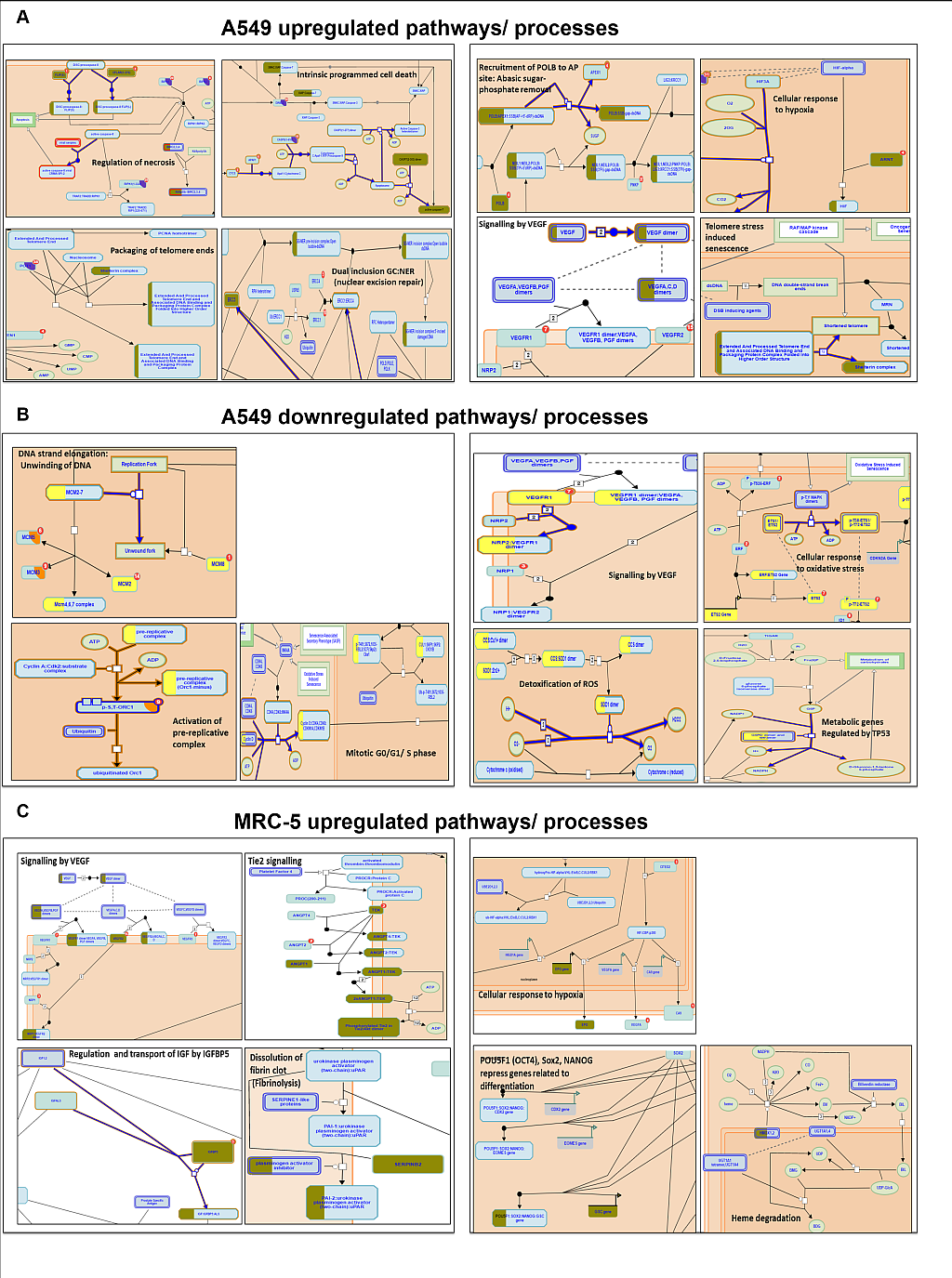

Supplement: S4 Fig — Pathway diagrams were constructed using the REACTOME pathway analysis software. Pathway diagrams are a representation of steps or processes of pathways with interconnected molecular events. Unique genes with altered expression patterns between A549 and MRC-5 cells were submitted as the query list onto the REACTOME web portal. Pathways were enriched when a significant number of the query list genes were part of a particular pathway against the overall pathway genes. Each pathway was considered statistically enriched when the p < 0.05. The dark green colour represents genes with upregulated expression levels while the bright yellow colour represents downregulated genes in a step or process. In A549 lung cancer cells, the combined treatment upregulated genes involved in (A); regulation of necrosis (p = 0.56E-5), intrinsic programmed cell death (p = 2.22E-2), packaging of telomere ends (p = 1.9E-2), dual inclusion GC:NER (p = 2 E-3), recruitment of POLB to AP site: abasic sugar-phosphate removal (p = 1.44E-2), cellular response to hypoxia (p = 1.19E-1), signaling by VEGF (p = 6.26E-1), and telomere stress induced senescence (p = 4.46E-2). Furthermore, in A549 lung cancer cells, the combined treatment downregulated genes involved in (B); DNA strand elongation: unwinding of DNA (p = 7.53E-6), activation of pre-replicative complex (p = 6.66E-5), mitotic G0/G1/S phase (p = 6.21E-4), signaling by VEGF (p = 3.31E-1), cellular response to oxidative stress (p = 5.86E-4), detoxification of ROS (p = 1.44E-3), and metabolic genes regulated by TP53 (p = 2.35E-2). In MRC-5 normal lung fibroblast cells, the combined treatment upregulated genes involved in (C); signaling by VEGF (p = 3.02E-4), Tie2 signaling (p = 3.38E-2), regulation and transport of IGF by IGFBP5 (p = 3.61E-2), Dissolution of fibrin clot (fibrinolysis) (p = 3.42E-2),cellular response to hypoxia (p = 1.32E-2), POU5F1 (OCT4), S0x2, NANOG repress genes related to differentiation (p = 1.72E-2), and heme degradation (p = [file pone.0182789.s004.tif]
