# Supplementary material for: Methyl pyruvate protects a normal lung fibroblast cell line from irinotecan-induced cell death: Potential use as adjunctive to chemotherapy
Source: PLoS One. 2017 Aug 10;12(8):e0182789. doi: 10.1371/journal.pone.0182789 (PMC5552298; doi:10.1371/journal.pone.0182789)
Supplement: S1 Table — (PDF) [file pone.0182789.s005.pdf]

Supplementary Table 1. Primer sets of each gene to be amplified

**Forward primer**	**Reverse primer**	**Annealing Temperatures (°C)**
GAPDH 5'ACACTCAGACCCCCACCACA 3'	GAPDH 5' CATTAGGCCCCTCCCCTCTT 3'	56
p53 5'TCAGATCCTAGCGTCGAGCCCCCTCT 3'	p53 5'CCCAGACGGAAAACCGTAGCTGCCT 3'	68
MDM2 5' ATCAGGCAGGGGAGAGTGAT 3'	MDM2 5' TCTACATACTGGGCAGGGC 3'	60
p21 5' ATGTCAGAACCGGCTGGGGA 3'	p21 5' GCCGTTTTCGACCCTGAGAG 3'	58
Caspase 3 5' AGAACTGGACTGTGGCATTGAG 3'	caspase 3 5' GCTTGTCGGCATACTGTTTCA 3'	59
BID 5' GCTGTATAGCTGCTTCCAGTG 3'	BID 5' GCTATCTTCCAGCCTGTCTTCTC 3'	61
Bax 5' CTGCAGAGGATGATTGCCG 3'	Bax 5' TGCCACRCGGAAAAAGACCT	58
Caspase 9 5' GCTCTTCTTTGTTCATCTCC 3'	Caspase 9 5' CATCTGGCTCGGGGTTACTGC 3'	60
