# Supplementary material for: Methyl pyruvate protects a normal lung fibroblast cell line from irinotecan-induced cell death: Potential use as adjunctive to chemotherapy
Source: PLoS One. 2017 Aug 10;12(8):e0182789. doi: 10.1371/journal.pone.0182789 (PMC5552298; doi:10.1371/journal.pone.0182789)
Supplement: S2 Table — (PDF) [file pone.0182789.s006.pdf]

Supplementary table 3: Statistical analysis (means ± SD) of the cell cycle phases for untreated and treated cells

**Cell lines**	**Cell cycle phases**	**% of cells at cell cycle phase**									
		**UN**	**4 IR**	**24 IR**	**48 IR**	**4 MP**	**24 MP**	**48 MP**	**4 COT**	**24 COT**	**48 COT**
**RMG1**	**Sub G0/G1**	3.3 ± 0.2	12.2 ± 0.2	13.2 ± 0.2	26.0 ± 0.1	1.3 ± 0.1	6.5 ± 0.1	19.3 ± 0.2	5.1 ± 0.1	5.2 ± 0.1	51.2 ± 0.2
	**G0/G1**	83.4 ± 0.2	71.0 ± 0.1	79.8 ± 0.1	33.3 ± 0.3	2.3 ± 0.2	88.3 ± 0.1	77.5 ± 0.1	75.2 ± 0.2	9.1 ± 0.2	46.8 ± 0.1
	**S**	12.5 ± 0.1	12.0 ± 0.1	6.1 ± 0.2	38.0 ± 0.2	88.2 ± 0.2	4.3 ± 0.1	4.3 ± 0.1	17.3 ± 0.2	40.0 ± 0.1	2.1 ± 0.2
	**G2/M**	0.8 ± 0.1	8.8 ± 0.3	1.0 ± 0.3	0.3 ± 0.1	4.8 ± 0.2	1.0 ± 0.2	2.4 ± 0.1	2.3 ± 0.2	46.0 ± 0.2	0.2 ± 0.1
**A549**	**Sub G0/G1**	0.5 ± 0.1	12.5 ± 1.0	69.2 ± 0.5	37.3 ± 1.0	13.5 ± 2.0	70.2 ± 1.5	36.5 ± 0.3	24.1 ± 0.1	52.7 ± 0.3	90.1 ± 0.1
	**G0/G1**	90.7 ± 0.1	79.1 ± 1.0	29.1 ± 1.0	56.4 ± 1.0	77.1 ± 3.0	28.6 ± 1.5	57.1 ± 0.3	69.5 ± 0.1	44.4 ± 0.3	9.2 ± 0.2
	**S**	5.2 ± 0.2	6.9 ± 0.2	1.9 ± 0.2	4.0 ± 0.1	7.0 ± 0.5	1.9 ± 0.2	4.0 ± 0.1	5.5 ± 0.2	2.7 ± 0.2	1.0 ± 0.1
	**G2/M**	3.7 ± 0.2	2.5 ± 0.2	0.3 ± 0.2	2.8 ± 0.1	2.2 ± 0.5	0.3 ± 0.2	2.9 ± 0.1	1.2 ± 0.2	0.3 ± 0.2	0.1 ± 0.1
**MDA-MB 231**	**Sub G0/G1**	3.6 ± 0.1	17.1 ± 1.0	41.5 ± 0.1	36.9 ± 0.1	16.3 ± 0.2	41.6 ± 0.2	36.9 ± 0.1	4.4 ± 0.2	7.1 ± 0.1	35.7 ± 0.1
	**G0/G1**	70.3 ± 0.1	44.9 ± 1.0	54.8 ± 0.2	51.9 ± 0.1	45.9 ± 0.1	54.3 ± 0.3	51.9 ± 0.1	51.1 ± 0.2	79.7 ± 0.1	34.8 ±0.1
	**S**	16.2 ± 0.2	29.7 ± 0.1	2.8 ± 1.0	7.5 ± 0.1	30.1 ± 1.0	2.4 ± 0.1	7.5 ± 0.1	34.1 ± 0.1	10.7 ± 0.1	20.1 ± 0.1
	**G2/M**	9.9 ± 0.2	15.3 ± 0.1	1.0 ± 1.0	3.9 ± 0.1	14.9 ± 1.0	1.4 ± 0.1	3.9 ± 0.1	10.5 ± 0.1	2.6 ± 0.2	19.4 ± 0.1
**MRC-5**	**Sub G0/G1**	1.2 ± 0.2	6.6 ± 0.3	3.9 ± 0.3	6.5 ± 0.3	6.6 ± 0.3	3.7 ± 0.1	6.3 ± 0.1	4.2 ±0. 2	4.2 ± 0.2	6.2 ± 0.2
	**G0/G1**	91.6 ± 2.0	80.1 ± 0.1	80.1 ± 0.3	74.9 ± 0.3	82.7 ± 2.5	80.3 ± 0.1	75.1 ± 0.1	82.5 ± 0.2	82.5 ± 0.2	88.1 ± 0.3
	**S**	8.6 ± 0.1	9.9 ± 0.1	13.4 ± 0.2	11.2 ± 0.1	10.0 ± 0.3	13.3 ± 0.2	11.5 ± 0.4	12.5 ± 0.2	11.5 ± 1.5	5.5 ± 0.2
	**G2/M**	0.4 ± 0.1	0.7 ± 0.2	2.8 ± 0.2	2.6 ± 0.1	0.6 ± 0.3	2.8 ± 0.2	2.2 ± 0.4	0.7 ± 0.2	0.5 ± 0.2	0.4 ± 0.2
**MRC-5 (Recovery)**	**Sub G0/G1**	1.1 ± 0.1	5.5 ± 0.3	6.1 ± 0.1	5.9 ± 0.3	5.3 ± 0.1	6.3 ± 0.3	5.9 ± 0.2	1.1 ± 0.1	9.3 ± 0.2	1.3 ± 0.1
	**G0/G1**	94.1 ± 0.1	85.0 ± 0.3	76.6 ± 0.2	65.0 ± 0.3	85.1 ± 0.1	76.1 ± 0.3	65.0 ± 0.2	84.3 ± 0.1	80.9 ± 0.2	56.4 ± 0.1
	**S**	4.3 ± 0.1	9.0 ± 0.3	15.7 ± 0.3	26.0 ± 0.2	8.9 ± 0.2	15.7 ± 0.3	26.0 ± 0.2	13.9 ± 0.1	9.2 ± 0.2	30.6 ± 0.3
	**G2/M**	0.6 ± 0.1	0.6 ± 0.3	1.7 ± 1.5	3.3 ± 0.2	0.7 ± 0.2	3.0 ± 0.3	3.2 ± 0.2	0.7 ± 0.1	0.7 ± 0.2	11.7 ± 0.3

UN – Untreated IR – Irinotecan **MP** – Methyl pyruvate **COT** – Cotreatment
