# Supplementary material for: Methyl pyruvate protects a normal lung fibroblast cell line from irinotecan-induced cell death: Potential use as adjunctive to chemotherapy
Source: PLoS One. 2017 Aug 10;12(8):e0182789. doi: 10.1371/journal.pone.0182789 (PMC5552298; doi:10.1371/journal.pone.0182789)
Supplement: S3 Table — (A) 13 upregulated MRC-5 genes (B) 17 upregulated A549 genes (C) 18 upregulated A549 genes. (PDF) [file pone.0182789.s007.pdf]

Supplementary Table 2

a. 13 upregulated MRC5 genes

Gene	Full name	Function	Ref
EPO	Erythropoietin/thrombopoietin	Retards DNA breakdown (promotes DNA repair) and prevents apoptosis in erythroid progenitor cells	[1]
GADD45G	Growth arrest DNA-damage-inducible protein 45	Generally, regulates cell cycle progression, genomic stability, DNA repair, and other stress-related responses.	[2, 3]
FASLG	(also known as *TNFSF6/CD95L*),	Initiation of extrinsic apoptotic pathway	[4]
GSC	Goosecoid	Embryonic development and control glucose metabolism through the regulation of multiple target genes in the liver, pancreas, and adipose tissue.	[5]
SOX10	SRY-box 10	Embryonic development and determination of cell fate.	[6]
KDR (VEGFR-2)	Vascular Endothelial Growth Factor	Angiogenesis	
TEK	TEK tyrosine kinase, endothelial	Angiogenesis. Cell survival	[7]
IGFBP5	Insulin growth factor-binding protein	Modulates cell survival, differentiation and apoptosis	[8]
SERPINFI	Serine protease inhibitor F1	Angiogenesis	
ANGPT1	Angiopoietin-1	Angiogenesis. Is a ligand for TEK	[9, 10]
PGF	Placenta growth factor (vascular endothelial growth factor-related protein).	Angiogenesis	[11]
HMOX1	Heme_oxygenase	Inflammatory and stress response.	[12]
SERPINB2	Serine Protease inhibitor 2 (Also - Plasminogen Activator Inhibitor-2)	Cell differentiation, tissue growth and regeneration.	[13]
		Regulation of Adaptive Immunity	[14]

b. 17 upregulated A549 genes

Gene	Full name	Function	Ref
BIRC3	Baculoviral IAP Repeat Containing 3	Negative regulator of non-canonical NF⍰B signalling.	[15]
ACSL4	Acyl-CoA synthetase long-chain family member 4	Essential in fatty acid metabolism	[16]
DDIT3	'DNA damage inducible transcript 3' (Formerly CHOP)	Cell survival and differentiation	[17]
ANGPT2	angiopoietin-2	Anti-angiogenic. A natural antagonist for Tie2 that disrupts in vivo angiogenesis.	[10, 18]

VEGFC	Vascular endothelial growth factor-FC	Promotes migration of endothelial cells. Is a ligand for the Flt4 (VEGFR-3) and KDR (VEGFR-2) receptor tyrosine kinases	[19]
CASP7	Caspase 7	Apoptosis	
CFLAR	CASP8 and FADD-like apoptosis regulator	the anti-apoptotic protein	[20]
ARNT/HIF-[illegible]	aryl hydrocarbon nuclear translocator	Control of glucose metabolism	[21]
PPP1R15A	phosphoprotein phosphatase regulatory subunit 15A also GADD34	Recovery from Endoplasmic Reticulum stress	[22, 23]
TNKS2	tankyrase-2	Tankyrases interact with the telomere reverse transcriptase complex (TERT)	
TERF2IP	Telomeric repeat binding factor 1	Shelterin complex member. Protect chromosomes against genomic instability	[24]
TINF2	TRF1 interacting nuclear factor 2	Part of the Shelterin complex	[25, 26]
SNAI2	Snail	Anti-apoptotic activity. Also influences metastasis.	[27, 28]
ERCC5	excision repair cross-species complementation5	Nucleotide excision repair	
POLB	DNA polymerase beta	Mediates the efficacy of chemotherapy through DNA repair machinery	[29]
BMI1	polycomb group gene	regulates proliferation of both normal and leukemic stem cells	[30]
DDB2	Damage specific DNA protein 2	*Xeroderma pigmentosum* gene – transcriptionally activated by p53. enhances global genomic repair of cyclobutane pyrimidine dimers and suppresses UV-induced mutagenesis.	[31]

c. **18 downregulated A549 genes**

Gene	Full name	Function	Reference
CDC20	Cell division cycle 20	Mitosis. Cell cycle checkpoint	[32]
MKI67	Antigen identified by monoclonal antibody ki-67	Proliferation marker antigen	[33]
FLT1/VEGR1	Vascular endothelial growth factor receptor 1	Angiogenesis	[19]
HMOX1	Heme_oxygenase	Inflammatory and stress response.	[12]

G6PD	Glucose-6-phosphate dehydrogenase	Glycolysis. Links to the pentose phosphate pathway	[34]
MCM2	Minichromosome maintenance complex component 2	Initiation of replication	[35]
ANGPT1	Angiopoietin-1	Angiogenesis. Is a ligand for TEK	[9, 10]
STMN1	Stathmin 1	It is thus a key protein in cell cycle progression. Inhibition of expression results in reduced cellular proliferation and accumulation of cells in the G_2/M phases of the cell cycle.	[36]
PINX1	PIN1/TERF1 interacting, telomerase inhibitor 1	Inhibits telomerase activity	[37]
FOXC2	Forkhead box C2	Immune response	[38]
AURKA	Aurora kinase 1	Mitosis: centrosome maturation and chromosome segregation	[39]
SOD1	Superoxide dismutase 1	Controls oxidative stress	[40]
COX5A	Cytochrome c oxidase subunit Va	Oxidative phosphorylation	[41]
DKC1	Dyskeratosis congenital 1, dyskerin	rRNA biogenesis	[42]
CCND3	Cyclin D3	Cell proliferation and differentiation	[43]
ETS2	Transcription factor (ETS protein family)	Cell cycle control	[44]
SKP2	S-phase kinase-associated protein 2, E3 ubiquitin protein ligase	Cell cycle control	[45]
SERPINF1	Serpin peptidase inhibitor clade F, member 1	Strongly inhibits angiogenesis	[46, 47]

References

1. Koury, M.J. and M.C. Bondurant, *Erythropoietin retards DNA breakdown and prevents programmed death in erythroid progenitor cells.* Science, 1990. **248**(4953): p. 378.
2. Na, Y.K., et al., *Hypermethylation of growth Arrest DNA-damage-inducible gene 45 in non-small cell lung cancer and its relationship with clinicopathologic features.* Molecules and Cells, 2010. **30**(1): p. 89-92.
3. Vairapandi, M., et al., *GADD45b and GADD45g are cdc2/cyclinB1 kinase inhibitors with a role in S and G2/M cell cycle checkpoints induced by genotoxic stress.* Journal of Cellular Physiology, 2002. **192**(3): p. 327-338.
4. Wang, M., et al., *FAS and FAS ligand polymorphisms in the promoter regions and risk of gastric cancer in Southern China.* Biochemical Genetics, 2009. **47**(7): p. 559-568.
5. Kang, K., et al., *Overexpression of goosecoid homeobox is associated with chemoresistance and poor prognosis in ovarian carcinoma.* Oncology Reports, 2014. **32**: p. 189-198.
6. Friedman, J.R. and K.H. Kaestner, *The Foxa family of transcription factors in development and metabolism.* Cellular and Molecular Life Sciences CMLS, 2006. **63**(19): p. 2317-2328.
7. Jones, N., et al., *Identification of Tek/Tie2 binding partners. Binding to a multifunctional dockinmg site mediates cell survival amd migration.* Journal of Biological Chemistry, 1999. **274**(43): p. 30896-30905.
8. Beattie, J., et al., *Insulin-like growth factor-binding protein-5 (IGFBP-5): a critical member of the IGF axis.* Biochemical Journal, 2006. **395**(Pt 1): p. 1-19.
9. Cheung, A.H., R.J. Stewart, and P.A. Marsden, *Endothelial Tie2/Tek ligands angiopoietin-1 (ANGPT1) and angiopoietin-2 (ANGPT2): Regional localization of the human genes to 8q22.3–q23 and 8p23.* Genomics, 1998. **48**(3): p. 389-391.
10. Asahara, T., et al., *Tie2 receptor ligands, angiopoietin-1 and angiopoietin-2, modulate VEGF-induced postnatal neovascularization.* Circulation Research, 1998. **83**(3): p. 233.
11. Maglione, D., et al., *Two alternative mRNAs coding for the angiogenic factor, placenta growth factor (PlGF), are transcribed from a single gene of chromosome 14.* Oncogene, 1993. **8**(4): p. 925-931.
12. Maines, M.D., *Heme oxygenase: function, multiplicity, regulatory mechanisms, and clinical applications.* The FASEB Journal, 1988. **2**(10): p. 2557-68.
13. Croucher, D.R., et al., *Revisiting the biological roles of PAI2 (SERPINB2) in cancer.* Nature Reviews Cancer, 2008. **8**(7): p. 535-545.
14. Schroder, W.A., et al., *A physiological function of inflammation-associated serpinB2 Is regulation of adaptive immunity.* The Journal of Immunology, 2010. **184**(5): p. 2663-2670.
15. Sun, S.-C., *Non-canonical NF-[kappa]B signaling pathway.* Cell Research, 2011. **21**(1): p. 71-85.
16. Mercade, A., A. Sanchez, and J.M. Folch, *Assignment of the acyl-CoA synthetase long-chain family member 4 (ACSL4) gene to porcine chromosome X.* Animal Genetics, 2005. **36**(1): p. 76-76.
17. Pereira, R.C., A.M. Delany, and E. Canalis, *CCAAT/Enhancer binding protein homologous protein (DDIT3) induces osteoblastic cell differentiation.* Endocrinology, 2004. **145**(4): p. 1952-1960.
18. Maisonpierre, P.C., et al., *Angiopoietin-2, a natural antagonist for Tie2 that disrupts in vivo angiogenesis.* Science, 1997. **277**(5322): p. 55.
19. Joukov, V., et al., *A novel vascular endothelial growth factor, VEGF-C, is a ligand for the Flt4 (VEGFR-3) and KDR (VEGFR-2) receptor tyrosine kinases.* The EMBO Journal, 1996. **15**(2): p. 290-298.
20. He, M.-X. and Y.-W. He, *CFLAR/c-FLIPL. A star in the autophagy, apoptosis and necroptosis alliance.* Autophagy, 2013. **9**(5): p. 791-793.
21. Zelzer, E., et al., *Insulin induces transcription of target genes through the hypoxia-inducible factor HIF-1α/ARNT.* The EMBO Journal, 1998. **17**(17): p. 5085.

22. Clavarino, G., et al., *Protein phosphatase 1 subunit Ppp1r15a/GADD34 regulates cytokine production in polyinosinic:polycytidylic acid-stimulated dendritic cells.* Proceedings of the National Academy of Sciences, 2012. **109**(8): p. 3006-3011.
23. KOJIMA, E., et al., *The function of GADD34 is a recovery from a shutoff of protein synthesis induced by ER stress: elucidation by GADD34-deficient mice.* The FASEB Journal, 2003. **17**(11): p. 1573-1575.
24. Aoude, L.G., et al., *Nonsense mutations in the Shelterin Complex genes ACD and TERF2IP in familial melanoma.* Journal of the National Cancer Institute, 2015. **107**(2).
25. Savage, S.A., et al., *TINF2, a component of the shelterin telomere protection complex, is mutated in Dyskeratosis Congenita.* The American Journal of Human Genetics, 2008. **82**(2): p. 501-509.
26. Simonsson, T., *The human TINF2 gene organisation and chromosomal localization.* Biochimie, 2001. **83**(5): p. 433-435.
27. Olmeda, D., et al., *Snai1 and Snai2 collaborate on tumor growth and metastasis properties of mouse skin carcinoma cell lines.* Oncogene, 2008. **27**(34): p. 4690-4701.
28. Peinado, H., D. Olmeda, and A. Cano, *Snail, Zeb and bHLH factors in tumour progression: an alliance against the epithelial phenotype?* Nature Reviews Cancer, 2007. **7**(6): p. 415-428.
29. Iwatsuki, M., et al., *A platinum agent resistance gene, POLB, is a prognostic indicator in colorectal cancer.* Journal of Surgical Oncology, 2009. **100**(3): p. 261-266.
30. Mohty, M., et al., *The polycomb group BMI1 gene is a molecular marker for predicting prognosis of chronic myeloid leukemia.* Blood, 2007. **110**(1): p. 380.
31. Tan, T. and G. Chu, *p53 binds and activates the Xeroderma Pigmentosum DDB2 gene in humans but not mice.* Molecular and Cellular Biology, 2002. **22**(10): p. 3247-3254.
32. Visintin, R., S. Prinz, and A. Amon, *CDC20 and CDH1: A Family of substrate-specific activators of APC-dependent proteolysis.* Science, 1997. **278**(5337): p. 460.
33. Schultz, I.J., et al., *Survivin and MKI67 mRNA expression in bladder washings of patients with superficial urothelial cell carcinoma correlate with tumor stage and grade but do not predict tumor recurrence.* Clinical Chemistry, 2006. **52**(7): p. 1440.
34. Cappellini, M.D. and G. Fiorelli, *Glucose-6-phosphate dehydrogenase deficiency.* The Lancet. **371**(9606): p. 64-74.
35. Ritzi, M., et al., *Human Minichromosome Maintenance proteins and human origin recognition complex 2 protein on chromatin.* Journal of Biological Chemistry, 1998. **273**(38): p. 24543-24549.
36. Rubin, C.I. and G.F. Atweh, *The role of stathmin in the regulation of the cell cycle.* Journal of Cellular Biochemistry, 2004. **93**(2): p. 242-250.
37. Cheung, D.H.-C., et al., *Nucleophosmin interacts with PIN2/TERF1-interacting telomerase inhibitor 1 (PinX1) and attenuates the PinX1 inhibition on telomerase activity.* Scientific Reports, 2017. **7**: p. 43650.
38. Coffer, P.J. and B.M.T. Burgering, *Forkhead-box transcription factors and their role in the immune system.* Nature Reviews Immunology, 2004. **4**(11): p. 889-899.
39. Bischoff, J.R., et al., *A homologue of Drosophila aurora kinase is oncogenic and amplified in human colorectal cancers.* The EMBO Journal, 1998. **17**(11): p. 3052.
40. Finkel, T. and N.J. Holbrook, *Oxidants, oxidative stress and the biology of ageing.* Nature, 2000. **408**(6809): p. 239-247.
41. Capaldi, R.A., *Structure and Function of cytichrome c oxidase.* Annu. Rev. Biochem., 1990. **59**: p. 569-596.
42. Heiss, N.S., et al., *Dyskerin localizes to the nucleolus and its mislocalization is unlikely to play a role in the pathogenesis of Dyskeratosis Congenita.* Human Molecular Genetics, 1999. **8**(13): p. 2515-2524.

43. Bartkova, J., et al., *Cyclin D3: requirement for G1/S transition and high abundance in quiescent tissues suggest a dual role in proliferation and differentiation.* Oncogene, 1998. **17**(8): p. 1027-1037.
44. Wasylyk, B., S.L. Hahn, and A. Giovane, *The Ets family of transcription factors*, in *EJB Reviews 1993*, P. Christen and E. Hofmann, Editors. 1994, Springer Berlin Heidelberg: Berlin, Heidelberg. p. 7-18.
45. Bai, C., et al., *SKP1 connects cell cycle regulators to the ubiquitin proteolysis machinery through a novel motif, the F-Box.* Cell, 1996. **86**(2): p. 263-274.
46. Law, R.H.P., et al., *An overview of the serpin superfamily.* Genome Biology, 2006. **7**(5): p. 216-216.
47. Dawson, D.W., et al., *Pigment epithelium-derived factor: A potent inhibitor of angiogenesis.* Science, 1999. **285**(5425): p. 245.
